# MetaGxData: Clinically Annotated Breast, Ovarian and Pancreatic Cancer Datasets and their Use in Generating a Multi-Cancer Gene Signature

**DOI:** 10.1038/s41598-019-45165-4

**Published:** 2019-06-19

**Authors:** Deena M. A. Gendoo, Michael Zon, Vandana Sandhu, Venkata S. K. Manem, Natchar Ratanasirigulchai, Gregory M. Chen, Levi Waldron, Benjamin Haibe-Kains

**Affiliations:** 1 Centre for Computational Biology, Institute of Cancer and Genomic Sciences, University of Birmingham, Birmingham, B15 2TT United Kingdom; 2 Princess Margaret Cancer Center, University Health Network, Toronto, M5G 2C1 Canada; 3 Department of Medical Biophysics, University of Toronto, Toronto, M5S 3H7 Canada; 4 Department of Biomedical Engineering, McMaster University, Toronto, L8S 4L8 Canada; 5 Institut Universitaire de Cardiologie et de Pneumologie de Québec, Université Laval, Québec City, G1V 4G5 Canada; 6 Graduate School of Public Health and Health Policy, Institute of Implementation Science in Population Health, City University of New York School, New York, 11101 USA; 7 Department of Computer Science, University of Toronto, Toronto, M5T 3A1 Canada; 8 Ontario Institute of Cancer Research, Toronto, M5G 0A3 Canada; 9 Vector Institute, Toronto, M5G 1M1 Canada

**Keywords:** Prognostic markers, Data integration

## Abstract

A wealth of transcriptomic and clinical data on solid tumours are under-utilized due to unharmonized data storage and format. We have developed the *MetaGxData* package compendium, which includes manually-curated and standardized clinical, pathological, survival, and treatment metadata across breast, ovarian, and pancreatic cancer data. *MetaGxData* is the largest compendium of curated transcriptomic data for these cancer types to date, spanning 86 datasets and encompassing 15,249 samples. Open access to standardized metadata across cancer types promotes use of their transcriptomic and clinical data in a variety of cross-tumour analyses, including identification of common biomarkers, and assessing the validity of prognostic signatures. Here, we demonstrate that *MetaGxData* is a flexible framework that facilitates meta-analyses by using it to identify common prognostic genes in ovarian and breast cancer. Furthermore, we use the data compendium to create the first gene signature that is prognostic in a meta-analysis across 3 cancer types. These findings demonstrate the potential of *MetaGxData* to serve as an important resource in oncology research, and provide a foundation for future development of cancer-specific compendia.

## Introduction

Ovarian, breast and pancreatic cancers are among the leading causes of cancer deaths among women, and recent studies have identified biological and molecular commonalities between them^1–4^. These cancers are part of hereditary syndromes related to mutations in a number of shared susceptibility genes that contribute to their carcinogenesis, including *BRCA1* and *BRCA2*^3,5^. As evidenced by epidemiological and linkage analysis studies, mutations and allelic loss in the *BRCA1* locus confers susceptibility to ovarian, pancreatic and early-onset breast cancer^5–8^. The *BRCA2* gene appears to account for a proportion of early-onset breast cancer that is roughly equal to that resulting from *BRCA1*^5,8^. *BRCA2*-mutation carriers with mutations within the ovarian cancer cluster region have been observed to exhibit greater risk for ovarian cancer^5^. In addition to common susceptibility genes, both tumours may express a variety of common biomarkers that include hormone receptors, epithelial markers (e.g., cytokeratin 7, Ber-EP4), growth factor receptors (Her2/neu) and other surface molecules^3^.

Commonalities between breast, ovarian, and pancreatic cancers have been observed not only for specific susceptibility genes, but at system-wide levels as well. In particular, molecular profiling across transcriptomes, copy-number landscapes, and mutational patterns emphasize strong molecular commonalities between basal-like breast tumours, high-grade serous ovarian cancer (HG-SOC), and basal-like pancreatic adenocarcinomas (PDACs)^2,9,10^. The growing list of parallels between Basal-like breast cancer, HG-SOC and basal-like PDACs include high frequency of *TP53* mutations and *TP53* loss, chromosomal instability, and widespread DNA copy number changes^2,9–11^. Statistically significant subsets of both Basal-like breast tumors and HG-SOC also share *BRCA1* inactivation, *MYC* amplification, and highly correlated mRNA expression profiles^2,9^. Subtype-specific prognostic signatures also reveal strong similarities between prognostic pathways in basal-like cancer and ovarian cancer, while ER-negative and ER-positive breast cancer subtypes exhibit different prognostic signatures^12^. These ongoing studies promote identification of shared prognostic and predictive biomarkers across multiple cancer subtypes for future treatment.

Continuous growth of publicly available databases of breast, ovarian and pancreas genome-wide profiles necessitates the development of large-scale computational frameworks that can store these complex data types, as well as integrate them for meta-analytical studies. Current bioinformatics initiatives provide extensive data repositories for microarray data retrieval and annotation of specific tumour types. These resources enable analysis of single datasets, but do not provide sufficient standardization across independent studies of single or multiple cancer types^13–19^ that are necessary for meta-analysis or other holistic analyses. This poses a challenge for meta-analytical investigations that aim to address global patterns across multiple forms of cancer, including for example, building multi-cancer gene signatures that generalize to new data^9,20,21^. Identifying robust prognostic signatures from transcriptomic data remains a major obstacle^9,12,21^, and requires large sample sizes that can only be provided by large-scale meta-analysis^20,22–26^. Additionally, most gene signatures derived from a single or small set of datasets are not generalizable to new data. In our recent systematic validation of ovarian signatures, primarily built from single datasets, we demonstrated that the concordance index of the best ovarian signatures only ranged from 0.54 to 0.58^27^, whereas signatures trained by meta-analysis could improve significantly on this performance^28^. The resulting standardized database of ovarian cancer profiles^29^ enabled numerous subsequent meta-analyses and the development of statistical methodology. Efforts to standardize analyses of the transcriptomes of multiple cancer types have focused on coupling microarray repositories with graphical user interfaces to allow researchers to address targeted biologic questions on collective transcriptome datasets^30–32^; however, these tools lack the generality to apply novel and potentially complex analyses.

An integrative framework is thus needed to harness the breadth of transcriptomic and clinical data from multiple cancer types, and to serve as a resource for integrative analysis across these aggressive cancer types. There are growing efforts towards the development of curated and clinically relevant microarray repositories for breast cancer, ovarian cancer, and pancreatic cancer data^4,29,33–36^. These studies provide a solid foundation for the development of a controlled language for clinical annotations and standardized transcriptomic data representation across the three cancer types. Here, we have developed the *MetaGxData* package compendium, which includes manually-curated and standardized clinical, pathological, survival, and treatment metadata for breast, ovarian, and pancreatic cancer transcriptome data. *MetaGxData* is the largest, standardized compendium of breast, ovarian and pancreas microarray datasets to date, spanning 86 datasets and encompassing 15,249 samples. Standardization of metadata across these cancer types promotes the use of their expression and clinical data in a variety of cross-tumour analyses, including identification of common biomarkers, establishing patterns of common co-expressed genes across cancer types, assessing the validity of prognostic signatures, and identification of new consensus signatures that reflects upon common biological mechanisms. In this paper, we present our flexible framework, unified nomenclature, as well as applications that demonstrate the analytical power of integrative analysis of a large number of breast, ovarian, and pancreatic cancer transcriptome datasets. As an example of its application, we integrated breast and ovarian cancer data to develop a multi-cancer gene signature and assessed its prognostic value in pancreatic cancer, demonstrating the existence of a multi-cancer prognostic gene signature.

## Results

### MetaGxData characterization and curation

The *MetaGxData* compendium integrates three packages containing curated and processed expression datasets for breast (*MetaGxBreast*), ovarian (*MetaGxOvarian*), and pancreatic (*MetaGxPancreas*) cancers. Our current framework extends upon the standardized framework we had already generated for curatedOvarianData^29^. Our proposed enhancements facilitate rapid and consistent maintenance of our data packages as newer datasets are added, and provides enhanced user-versatility in terms of data rendering across single or multiple datasets. All of these datasets can be downloaded through the MetaGxBreast, MetaGxOvarian and MetaGxPancreas R data packages publicly available through the Bioconductor ExperimentHub^37–39^. Vignettes outlining how to access the MetaGxBreast, MetaGxOvarian and MetaGxPancreas datasets in R are available through the Bioconductor website.

**Figure 1:**
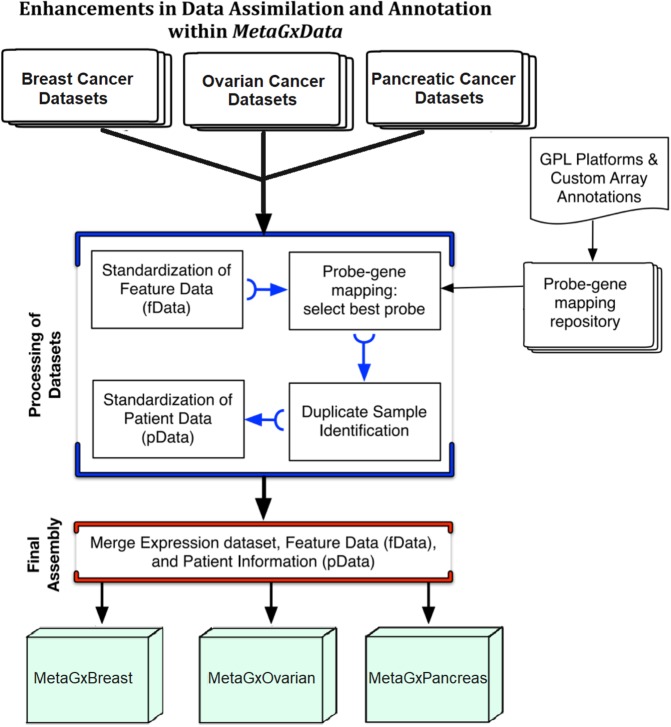
Diagrammatic representation of the data processing pipeline for packages that are part of the MetaGxData compendium. Depicted are the processes involved in downloading a dataset, and standardization of molecular (gene) and clinical (patient) data to produce cancer-specific compendia that abide by the MetaGxData framework.

**Figure 2:**
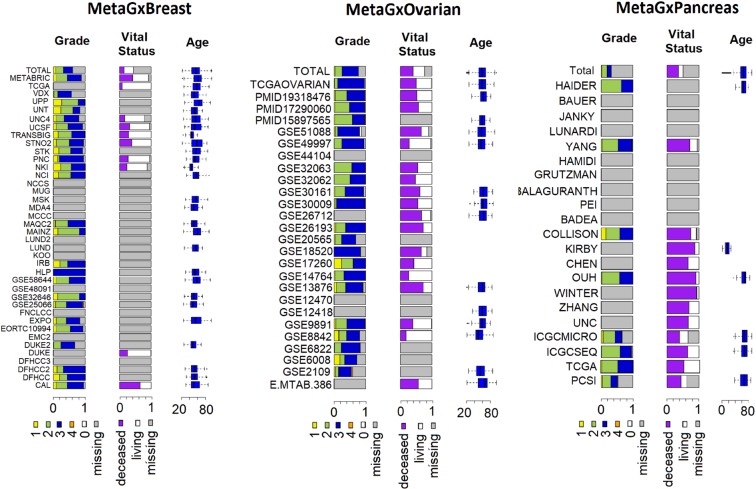
Schematic representation of some of the common clinical variables (pData) that are available across datasets in MetaGxBreast, MetaGxOvarian, and MetaGxPancreas. The Stacked bar plots indicate the percentage of samples in every dataset annotated with a particular variable designation. Continuous numeric values are represented by box plots.

We developed semi-automatic curation scripts to standardize gene and clinical annotations of our breast, ovarian and pancreatic cancer datasets based on the nomenclature used in The Cancer Genome Atlas (TCGA) **(**Supplementary File S1**)**^2,29^. At its core, the MetaGxData compendium represents a unified pipeline for processing datasets within a given form of cancer, and providing cancer-specific data packages to users with standardized gene and clinical annotations **(**Fig. 1**)**. Such annotations include a host of relevant categorical variables that reflect upon tumour histology (stage, grade, primary site, etc.), as well as categorical and numerical variables crucial for survival analysis and prognostication in these cancers (including overall survival, recurrence-free survival, distant-free survival, and metastasis-free survival) **(**Supplementary Fig. S2**)**. Most importantly, we have provided a number of comparable and overlapping clinicopathological features across breast, ovarian and pancreatic cancer samples, such as age at diagnosis, tumour grade, or vital status **(**Fig. 2**)**. Where some datasets lack vital status or other endpoints, we have included information on other endpoints, such as relapse free survival (breast and ovarian cancer datasets) and distant metastasis free survival (breast cancer datasets only). Additional common variables between the datasets can be seen in the supplementary figures **(**Supplementary Figs S3–S5**)**. We also provide tumour-specific and critical annotations for each tumour type, including, for example, biomarker identification status (HER2, ER, PR) in breast cancer, and TNM status for pancreatic datasets. Treatment information across the cancers is provided when available.

For subsequent analyses presented in this work, overall survival was used as the primary endpoint, and datasets lacking vital status were excluded from the analysis. For pancreatic cancer, survival information was obtained exclusively using overall survival as the primary endpoint.

### Analysis of prognostic genes in breast, ovarian, and pancreatic cancer

**Figure 3:**
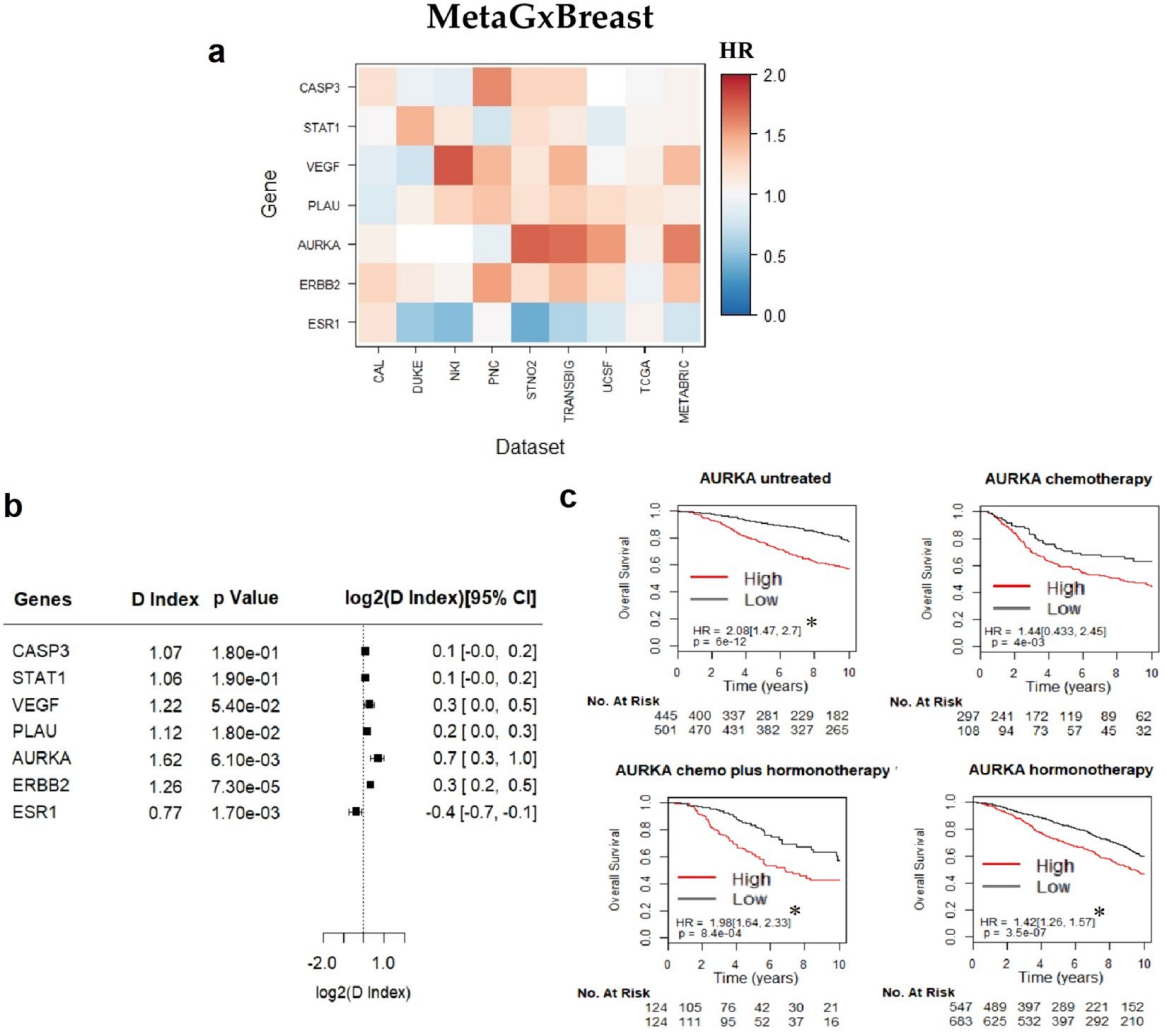
Assessment of the prognostic value of seven key gene modules in breast cancer, using the MetaGxBreast package. (**a**) Heatmap representation of hazard ratios for each gene module, across 9 datasets. The estimate is presented as a hazard ratio for each gene. Ratios greater than 1 (red) indicate worse prognosis for elevated expression levels of that gene in the respective datasets. (**b**) Random effects meta-estimates of the hazard ratios for each gene, calculated by pooling the hazard ratios from each individual dataset. (**c**) Kaplan-Meier curves of the most prognostic gene with p < 0.05, in this case AURKA. Each KM plot represents patients of a specific treatment type. Within each plot, patients are split into ‘high’ and ‘low’ based on the median AURKA score.

**Figure 4:**
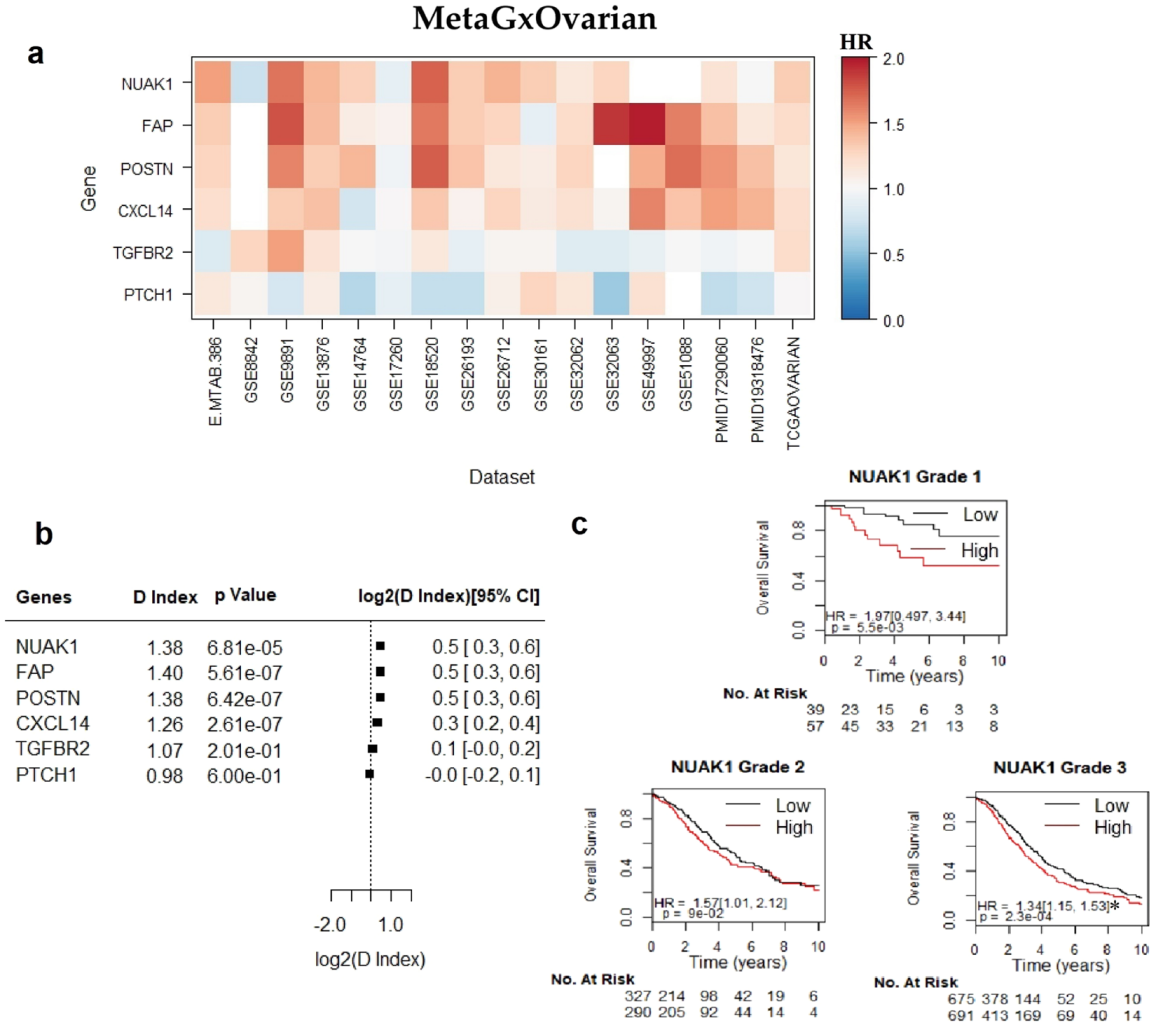
Assessment of the prognostic value of six key genes in ovarian cancer, using the MetaGxOvarian package. (**a**) Heatmap representation of hazard ratios for each gene, across 17 datasets. The estimate is presented as a hazard ratio for each gene. Ratios greater than 1 (red) indicate worse prognosis for elevated expression levels of that gene in the respective datasets. (**b**) Random effects meta-estimates of the hazard ratios for each gene, calculated by pooling the hazard ratios from each individual dataset. (**c**) Kaplan-Meier curves of NUAK1. Each KM plot represents patients of a specific tumour grade. Within each plot, patients are split into ‘high’ and ‘low’ based whether they fall above or below the median NUAK1 gene expression. The asterisks above the D indices indicate whether the D index was statistically significant (p < 0.05).

**Figure 5:**
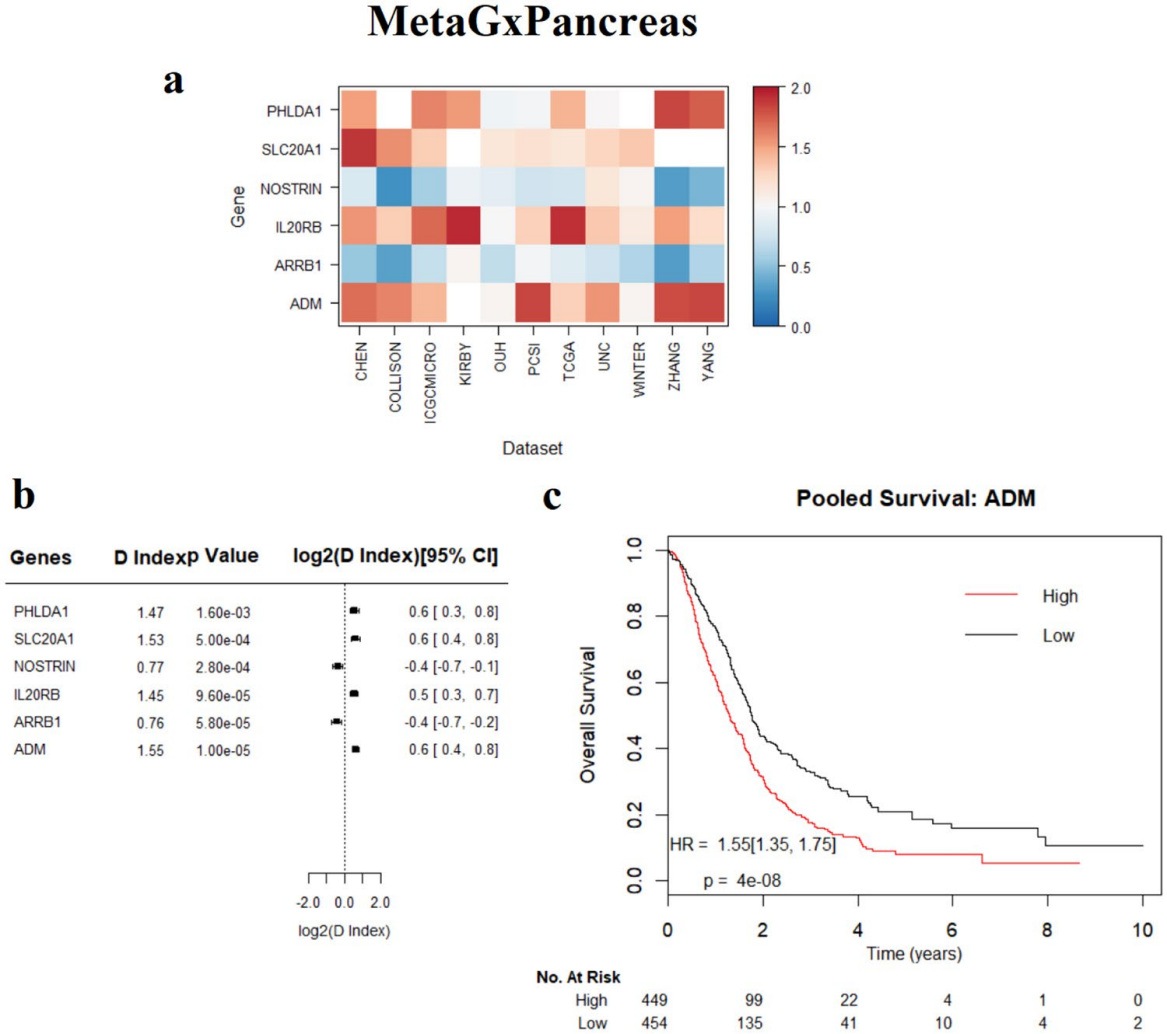
Assessment of the prognostic value of genes in pancreatic cancer, using the MetaGxPancreas package. (**a**) Heatmap representation of hazard ratios for each gene, across 11 datasets. The estimate is presented as a hazard ratio for each gene. Ratios greater than 1 (red) indicate worse prognosis for elevated expression levels of that gene in the respective datasets. (**b**) Random effects meta-estimates of the hazard ratios for each gene, calculated by pooling the hazard ratios from each individual dataset. (**c**) Kaplan-Meier curve of ADM.

The wealth and breadth of transcriptomic datasets in *MetaGxData* can be used as a framework for translational cancer research. As an example of the versatility of our packages, we conducted a meta-analysis of the prognostic value of well-studied prognostic genes in ovarian cancer and pancreatic cancer, as well as our previously published gene modules in breast cancer using the *MetaGxBreast*, *MetaGxPancreas* and *MetaGxOvarian* packages **(**Figs 3–5**)**^22,23,27,28^. A total of 6 ovarian genes (PTCH1, TGFBR2, CXCL14, POSTN, FAP, and NUAK1), 36 pancreas genes from the gene signature developed by Haider *et al*.^40^, and 7 breast cancer gene modules (ESR1, ERBB2, STAT1, CASP3, PLAU, VEGF, and AURKA) were tested. For breast cancer gene modules, each module is comprised of a set of highly-correlated genes (using Gram-Schmidt variable selection) relating to specific cancer biological processes that we previously demonstrated to have prognostic utility in breast cancer^23,28^. For simplicity, each module is identified by a standard ‘prototype gene’; as an example, the ‘AURKA’ module contains genes that are highly correlated with the proliferation gene AURKA **(**Fig. 3a**)**.

The hazard ratio of tested genes and gene modules was determined by calculating the D.index, which is an estimate of the log hazard ratio (HR) comparing two equal sized groups. We observed that the direction of hazard ratios of these genes (HR > 1 or HR < 1) was fairly consistent, largely deviated from HR = 1, and was statistically significant across datasets. Genes with hazard ratios closer to 1 demonstrated greater variability in the direction of the HR index across datasets, owing to their decreased prognostic relevance **(**Fig. 3a**)**. Furthermore, log rank tests were used to determine whether splits in the survival curves generated by using the genes to group patients into high and low score groups were statistically significant.

Unsurprisingly, higher gene expression levels of the proliferation gene AURKA indicate poorer survival in breast cancer (log rank p = 1.1e-16, n = 4,161) **(**Fig. 3c**)**. This supports previous findings regarding the importance of this gene in biology-driven signatures of breast cancer, and its comparable prognostic effect with other multi-gene prognostic signatures^22,23,35,41,42^. We have also observed that the NUAK1 gene exhibits worst prognosis in ovarian cancer (log rank p = 6.2e-9, n = 2,450) **(**Fig. 4c**)**. We have previously demonstrated the utility of NUAK1 in the development of a debulking signature that can predict the outcome of cytoreductive surgery^28^. Figure 5 demonstrates the results of the 6 top-most statistically significant genes from the Haider *et al*. pancreatic gene signature^40^. Of these genes, we have observed that adrenomedullin (ADM) exhibits the worst prognosis in pancreatic cancer **(**Fig. 5c**)**. High expression levels of ADM led to poor outcomes in patients, which is consistent with previous findings that ADM is over expressed in PDAC and enhances pancreatic cancer cell invasion^43^.

### Meta-analysis of gene expression prognosis across cancers

Our single-gene prognostic analysis can easily be extended to a genome-wide meta-analysis across individual cancer types, or combining several cancer types. To this end, we first determined the prognostic capability of 22,410 genes that are common across predominantly female cancers **(**Supplementary File S6**)**. We identified 30 genes that are significantly prognostic across both tumours (False Discovery Rate [FDR] < 5%). From this list of prognostic genes, we subsequently identified 12 genes that share same-direction hazard ratios in both breast and ovarian cancers: 3 genes have elevated expression values indicative of worse prognosis in both cancers (HR > 1), and 9 genes have better prognosis (HR < 1) **(**Supplementary File S6**)**. Such analyses can be used to test pan-cancer hypotheses across much larger sample sizes than previously possible, and will allow deeper study of relationships between cancer subtypes.

We additionally conducted a genome-wide analysis of all the genes present across the MetaGxPancreas datasets in order to identify highly prognostic genes (Supplementary File S6). Only genes present in at least 6 of the 12 datasets containing overall survival information were considered in the search for the most prognostic genes (n = 19,245 genes). The 3 genes that led to the poorest outcomes when overexpressed (largest HR) with FDR-adjusted p-values under 5% were FAM83A (HR = 1.83), HMGA2 (HR = 1.73), and KRT7 (HR = 1.72). The 3 genes whose expression was most indicative of better outcomes (smallest HR), with an FDR-adjusted p-values under 5% were PPP1R10 (HR = 0.69), FRZB (HR = 0.7), and GATA6 (HR = 0.71), and FAM189A2 (HR = 0.68). Notably, FAM189A2 was also identified in our analysis as the only gene that is indicative of worse outcome (FDR < 0.05, HR < 1) across breast, ovarian, and pancreatic cancers **(**Supplementary File S6**)**.

### MetaGx gene signature creation and prognosis in breast, ovarian and pancreatic cancer

We developed a gene signature that is prognostic in both breast and ovarian cancers by running a single-gene, genome-wide prognostic analysis on 22,410 genes as above, but excluding several large breast and ovarian datasets for use as validation cohorts. The METABRIC dataset (n = 2136 samples) from MetaGxBreast, and 5 of the largest ovarian datasets (GSE9891, GSE32062, GSE49997, GSE26712, GSE51088) were removed from the analysis for later use as the validation cohort to test the signature. Using only the training sets, meta-analysis identified 53 genes with significant hazard ratios in both cancers (FDR < 5%, HR > 1.125 or HR < 0.875), which were used to form the MetaGx signature **(**Table 1**)**. The direction of association of the genes comprising the signature was chosen based on the hazard ratios (HR > 1 positive direction). Notably, the MetaGx signature included 3 genes (DDB2, GSTZ1, and FAM1892A) that had been previously identified from the set of 12 genes sharing same-direction hazard ratios in the meta-analysis of breast and ovarian cancers **(**Supplementary File S6**)**.

**Table 1:** Genes present in the MetaGx gene signature.

	Gene Symbol	Description	Entrez ID	Direction
1	ACKR3	atypical chemokine receptor 3	57007	1
2	ACTN4	actinin alpha 4	81	1
3	ARHGAP21	Rho GTPase activating protein 21	57584	1
4	C12orf49	chromosome 12 open reading frame 49	79794	1
5	CACNB3	calcium voltage-gated channel auxiliary subunit beta 3	784	1
6	CAMK1D	calcium/calmodulin dependent protein kinase ID	57118	1
7	CAMSAP3	calmodulin regulated spectrin associated protein family member 3	57662	−1
8	CBFB	core-binding factor beta subunit	865	1
9	CDC37L1	cell division cycle 37 like 1	55664	−1
10	CDK19	cyclin dependent kinase 19	23097	1
11	CLDN4	claudin 4	1364	1
12	CMBL	carboxymethylenebutenolidase homolog	134147	1
13	COP1	COP1, E3 ubiquitin ligase	64326	1
14	CRABP2	cellular retinoic acid binding protein 2	1382	1
15	CSE1L	chromosome segregation 1 like	1434	1
16	DARS2	aspartyl-tRNA synthetase 2, mitochondrial	55157	1
17	DDB2	damage specific DNA binding protein 2	1643	−1
18	DPP4	dipeptidyl peptidase 4	1803	1
19	EGFR	epidermal growth factor receptor	1956	1
20	FAM189A2	family with sequence similarity 189 member A2	9413	−1
21	GSTZ1	glutathione S-transferase zeta 1	2954	−1
22	IMPDH1	inosine monophosphate dehydrogenase 1	3614	1
23	IRF3	interferon regulatory factor 3	3661	1
24	KATNAL1	katanin catalytic subunit A1 like 1	84056	1
25	KIF11	kinesin family member 11	3832	1
26	LATS2	large tumor suppressor kinase 2	26524	1
27	LOXL2	lysyl oxidase like 2	4017	1
28	MOCS1	molybdenum cofactor synthesis 1	4337	−1
29	MREG	melanoregulin	55686	−1
30	MSC	musculin	9242	1
31	MYADM	myeloid associated differentiation marker	91663	1
32	MYLK3	myosin light chain kinase 3	91807	−1
33	NAE1	NEDD8 activating enzyme E1 subunit 1	8883	1
34	NID2	nidogen 2	22795	1
35	OPRM1	opioid receptor mu 1	4988	1
36	PLAU	plasminogen activator, urokinase	5328	1
37	PPEF1	protein phosphatase with EF-hand domain 1	5475	1
38	PWP1	PWP1 homolog, endonuclein	11137	1
39	RALY	RALY heterogeneous nuclear ribonucleoprotein	22913	1
40	RARRES3	retinoic acid receptor responder 3	5920	−1
41	REX1BD	required for excision 1-B domain containing	55049	1
42	SERPINB2	serpin family B member 2	5055	1
43	SIPA1L2	signal induced proliferation associated 1 like 2	57568	1
44	STK3	serine/threonine kinase 3	6788	1
45	TERF2	telomeric repeat binding factor 2	7014	1
46	TEX261	testis expressed 261	113419	1
47	TGFBI	transforming growth factor beta induced	7045	1
48	TNFRSF18	TNF receptor superfamily member 18	8784	−1
49	TPD52L2	tumor protein D52 like 2	7165	1
50	UTP6	UTP6, small subunit processome component	55813	1
51	ZFAND2A	zinc finger AN1-type containing 2 A	90637	1
52	ZNF204P	zinc finger protein 204, pseudogene	7754	−1
53	ZSCAN32	zinc finger and SCAN domain containing 32	54925	−1

**Figure 6:**
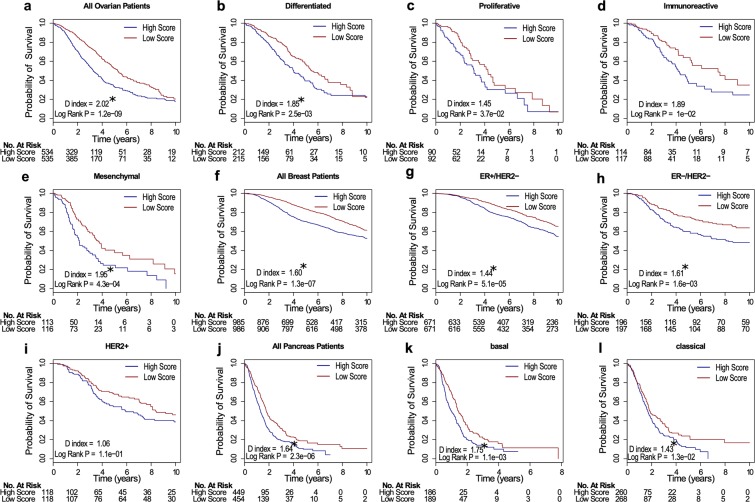
Survival curves for the MetaGx signature with patients stratified by molecular subtypes. (**a**–**e**) Survival curves in ovarian cancer. (**f**–**i**) Survival curves in breast cancer. (**j**–**l**) Survival curves in pancreatic cancer. The asterisks above the D indices indicate whether the D index was statistically significant (p < 0.05).

The top 5 signatures from our recent review of ovarian gene signatures were evaluated alongside the MetaGx signature, and each signature was tested in the molecular subtypes identified by The Cancer Genome Atlas Research Network (immunoreactive, proliferative, mesenchymal, differentiated subtypes)^1,27^. The MetaGx signature was the most prognostic of the ovarian signatures tested in an analysis containing all the patients (HR 2.02, n = 1,069) and was the only signature providing statistically significant prognostic capabilities within each subtype (log rank tests p < 0.05). Although the D index was prognostic in the differentiated subtype (HR 1.85, n = 427) and the most prognostic of the signatures tested in the Mesenchymal subtype (HR 1.95, n = 229), the MetaGx signature did not yield statistically significant D indices in the immunoreactive and proliferative subtypes **(**Fig. 6a–e**)**.

In breast cancer, the MetaGx signature was benchmarked against the clinically relevant mammaprint and oncotype DX signatures^44–46^. Our three gene (ER, HER2, and AURKA) subtype classification model (SCM) was chosen to classify patients into the ER+/HER2−, ER−/HER2−, and HER2+ subtypes^35^. The MetaGx signature was highly prognostic in the analysis using all patients (HR 1.60, n = 1,971) **(**Fig. 6f**)** and had the largest D index in the ER−/HER2− subtype (HR 1.61, n = 393) **(**Supplementary Fig. S7**)**.

We further tested the prognostic value of the MetaGx signature in pancreatic cancer and benchmarked it against pancreatic signatures from the literature. A signed average approach was implemented for evaluation, where the direction of association of the genes comprising the signature were chosen based on the hazard ratios (HR > 1 positive direction)^40,47–49^. Briefly, in each patient, genes from the signature whose expression led to poor outcomes (HR > 1) were added together, and genes whose expression led to a favorable prognosis (HR < 1) were subtracted. Accordingly, higher signature scores (ie, signed average) were associated with poorer outcomes. Information pertaining to the genes comprising each of the pancreatic signatures can be found in Supplementary File S8.

Of the 5 signatures tested, the MetaGx signature was the most prognostic in the analysis of all the patients (HR 1.64, n = 903) and was the only signature that yielded a statistically significant difference in survival within both the basal (log rank p = 1.1e-3, n = 375) and the classical (log rank p = 1.3e-2, n = 528) pancreatic cancer molecular subtypes identified by Moffitt *et al*. **(**Table 2, Fig. 6j–l**)**^50^.

**Table 2:** Prognostic value of Pancreatic Cancer Gene Signatures.

	Gene Signature - Subtype	D Index	D Index 95% CI	D Index P	Log Rank Test P
1	MetaGx - All Patients	1.64	(1.37, 1.90)	1.9e-04	2.3e-06
2	MetaGx - basal	1.75	(1.31, 2.19)	1.1e-02	1.1e-03
3	MetaGx - classical	1.43	(1.09, 1.77)	3.7e-02	1.3e-02
4	Newhook PLos one^a^ - All Patients	1.22	(0.97, 1.47)	1.1e-01	1.2e-01
5	Newhook PLos one^a^ - basal	1.01	(0.80, 1.23)	9e-01	7.9e-01
6	Newhook PLos one^a^ - classical	0.99	(0.77, 1.20)	9e-01	6.2e-01
7	Haider Gen Med^b^ - All Patients	1.56	(1.23, 1.88)	6.8e-03	4.7e-06
8	Haider Gen Med^b^- basal	1.22	(0.88, 1.57)	2.4e-01	2.2e-01
9	Haider Gen Med^b^ - classical	1.43	(1.15, 1.71)	1e-02	9.5e-02
10	Grutzmann Oncogene^c^ - All Patients	1.35	(1.22, 1.49)	1.3e-05	2.1e-06
11	Grutzmann Oncogene^c^ - basal	1.29	(0.91, 1.67)	1.7e-01	7.8e-03
12	Grutzmann Oncogene^c^ - classical	1.23	(1.01, 1.46)	6.2e-02	1.1e-01
13	Stratford PLos med^d^ - All Patients	1.39	(1.09, 1.68)	2.9e-02	6.3e-03
14	Stratford PLos med^d^ - basal	1.22	(1.01, 1.43)	6.4e-02	2.6e-01
15	Stratford PLos med^d^- classical	1.29	(0.94, 1.63)	1.4e-01	6.7e-02

^a^T. E. Newhook *et al*., A thirteen-gene expression signature predicts survival of patients with pancreatic cancer and identifies new genes of interest, PLoS One, vol. 9, no. 9, p. e105631, Sep. 2014.

^b^S. Haider *et al*., A multi-gene signature predicts outcome in patients with pancreatic ductal adenocarcinoma, Genome Med., vol. 6, no. 12, p. 105, Dec. 2014.

^c^R. Grutzmann *et al*., Meta-analysis of microarray data on pancreatic cancer defines a set of commonly dysregulated genes, Oncogene, vol. 24, no. 32, pp. 5079–5088, Jul. 2005.

^d^J. K. Stratford *et al*., A six-gene signature predicts survival of patients with localized pancreatic ductal adenocarcinoma, PLoS Med., vol. 7, no. 7, p. e1000307, Jul. 2010.

We determined the spearman correlation between patients signature scores and our gene modules in order to investigate the biological processes present in our signature **(**Supplementary Fig. S9**)**. In all 3 cancers, the signature scores had strong positive spearman correlations with the PLAU module (0.67 in pancreas, 0.40 in breast, 0.69 in ovarian) and relatively strong negative correlations with the ESR1 module (−0.51 in pancreas, −0.52 in breast, −0.35 in ovarian). Recent studies have shown that most published gene signatures often perform no better than 1,000 random signatures of equal length. To test this observation, the MetaGx signature was tested in the pancreatic cancer, ovarian cancer and breast cancer test datasets against 1,000 random signatures of equal size^51^. In all three cases, the magnitude of the hazard ratio from the MetaGx signature was larger than the random signatures’ hazard ratio (p = 0.001 for all three cancers) **(**Supplementary Fig. S10**)**.

## Discussion

Meta-analysis of multiple cancer types is an area of high interest, with ongoing research continually supporting the growing relationship between these malignancies and suggesting common patterns of tumour biology^52^. We provide an integrative, standardized, and comprehensive platform to facilitate analysis between breast, ovarian, and pancreatic cancer. This platform provides a flexible framework for data assimilation and unified nomenclature, with standardized data packages hosting the largest compendia of breast, ovarian, and pancreatic cancer transcriptomic and clinical datasets available to date.

Integration of genomic data into standardized frameworks is challenged by the inconsistency of the clinical curations across datasets and across tumour types. Annotation of clinicopathological variables may vary widely due to different protocols in different laboratories, institutions, and across international boundaries. We have standardized, as much as possible, the catalog of clinical variables within each tumour type. For characteristics pertaining to a specific tumour type, including ER, PGR, and HER2 IHC status in breast cancer samples, we have generated a semantic positive/negative variable to reflect IHC status. This facilitates searching across all patients irrespective of the original assay annotations that may have binary, numeric, or qualitative. Similarly, a binary variable has been assigned to ovarian cancer patients to reflect whether they had been treated with platinum, taxol, or neoadjuvant therapy. Many of the annotated variables (ex: stage and tumour grade in MetaGxOvarian) have also been standardized to facilitate comparisons across multiple studies. Further analyses using our previously developed packages (curatedOvarianData) have indicated good consistency across datasets, and ultimately facilitated uniform and consistent investigations on the prognostic effect of biomarkers in ovarian cancer survival^53,54^.

The scale of MetaGxData facilitates identification of gene signatures that are prognostic across multiple forms of cancer. Using this compendium, we developed a gene signature that is prognostic for breast, ovarian, and pancreatic cancers. Requiring genes to be prognostic across multiple datasets should help distinguish between general and disease-specific processes affecting patient survival, and allow signatures to generalize better to new datasets, as opposed to conventional signature creation methods that select genes based on cox proportional hazard models in a single dataset. We have demonstrated that the multi-cancer MetaGx signature outperformed the top ovarian signatures identified in our previous review in an analysis conducted on all patients with overall survival as the endpoint. It was also more prognostic than the clinically-relevant Mammaprint and OncotypeDX signatures in the ER−/HER2− breast cancer subtype, and more prognostic than pancreas-specific signatures in pancreatic cancer. Furthermore, it was the only signature that was prognostic in each molecular subtype of pancreatic cancer, and was highly prognostic in the basal-like subtype. Notably, the MetaGx signature was not prognostic in the HER2− breast subtype or the immunoreactive and proliferative ovarian subtypes. One possible explanation for this behavior is that the number patients with those subtypes are fewer, compared to the majority of patients that were used to as the training set. This is particularly true for the Her2− subtype in breast cancer (n = 236 Her2− patients, in a training set of n = 1,969 breast cancer patients). However, we are unaware of any gene signature to-date that is prognostic across each subtype based on a meta-analysis of multiple datasets. Indeed, the clinically used Mammaprint signature, as an example, is only used for ER+/Her2− patients.

The large number of datasets offered as part of MetaGxData provides researchers with the ability to select different datasets for their respective analyses. As such, it is conceivable that researchers may select particular datasets to highlight the significance of signatures. However, the magnitude of the samples and datasets provided by the compendium makes it arguably difficult for researchers to justify why some datasets have been retained and others dismissed. In the current literature, many existing publications have derived prognostic signatures based on a comparison of 3–5 datasets. With the release of the MetaGxData, researchers now need to develop signatures that harness the full compendium. Hopefully, this will result in the production of more rigorous signatures, as these signatures would need to be prognostic across an entire meta-analysis.

To our knowledge, the MetaGx signature represents the first signature demonstrated to be prognostic in a meta-analysis across three cancers. This includes pancreatic cancer, which had been selected as an independent validation set for testing the signature. Our signature predicts poor outcomes associated with metastases for patients, based on our observations that patients signature scores across all three cancers consistently had strong positive correlations with our PLAU tumor metastases module. Furthermore, since the signature was consistently negatively correlated with the ESR1 module in all three cancers, and high signature scores led to poor outcomes, we believe the signature also models the poor outcomes associated with increased ER pathway activity in patients. Our signature provides additional support for the role of CLDN4 in pancreas, breast and ovarian malignancies. Higher expression levels of this gene placed patients in the high score group that had poorer outcomes in all 3 of these cancers. This is in agreement with numerous studies that have shown CLDN4 to be overexpressed in pancreatic, ovarian, and breast tumors relative to normal tissue^55–59^. It is also interesting to observe that FAM189A2 was one of the top genes across all 3 cancer that was indicative of worse outcomes when expression levels were low (HR < 1), which is consistent with what has been shown in lung and thyroid cancer^60,61^.

In conclusion, the MetaGxBreast, MetaGxOvarian and MetaGxPancreas packages follow a unified framework that facilitates integration of oncogenomic and clinicopathological data. We have demonstrated how our packages facilitate easy meta-analysis of gene expression and prognostication in breast, ovarian and pancreatic cancer. We have also demonstrated that leveraging this data in meta-analysis can lead to gene signatures that outperform clinically relevant breast signatures in ER−/HER2− patients, and outperform ovarian signatures developed from single datasets, as well as a number of published pancreatic cancer signatures. These packages have the potential to serve as an important resource in oncology and methodological research and provide a foundation for future development of cancer-specific compendia.

## Methods

### Breast cancer data acquisition

Breast cancer datasets were extracted from our previous meta-analysis of breast cancer molecular subtypes, which includes 39 microarray datasets from a variety of commercially available microarray platforms published from 2002 to 2014^35^. Additional datasets were extracted from the Gene Expression Omnibus (GEO) and manually curated. Gene expression and clinical annotation for Metabric were downloaded from EBI ArrayExpress and combined into a dataset of 2,136 samples^62^. The cgdsr R package was used to extract 1,098 tumour samples from The Cancer Genome Atlas (TCGA), and matching clinical annotations for these samples were downloaded from the TCGA Data Matrix portal (https://tcga-data.nci.nih.gov/tcga/)^2,63^. Combining these studies produced a total of 39 breast cancer microarray expression datasets spanning 10,004 samples. Of these 10,004 samples, survival information is available for 6,847 patients, including overall survival (n = 4,425), metastasis free survival (n = 2,695), and relapse free survival (n = 1,858).

### Ovarian cancer data acquisition

Ovarian microarray expression datasets were obtained from our recent update of the curatedOvarianData data package, onto which we have added 5 expression datasets to the originally published version^29^, for a total of 26 microarray datasets spanning 3,526 samples. To obtain these datasets we first used the curatedOvarianData pipeline to generate the “FULLcuratedOvarianData” version of the package, which differs from the public version in that probe sets for same gene are not merged (https://bitbucket.org/lwaldron/curatedovariandata). Of the 3,526 samples, survival information is available for 2,726 patients, including overall survival (n = 2,712) and relapse free survival (n = 1,928).

### Pancreatic cancer data acquisition

Pancreatic ductal adenocarcinoma (PDAC) datasets were obtained by curating datasets available from the literature. A total of 21 datasets were curated for a total of 1,719 patient transcriptomic profiles. Of the 21 datasets, overall survival data was present for 12 studies. Consequently, of the 1,719 samples survival information is available for 1,000 patients, including overall survival (n = 1,000) and no relapse free survival data.

### Processing of gene expression datasets

The processing of breast and ovarian cancer microarray datasets was previously described^29,35^. The pancreatic cancer datasets were processed in the manner described within the original studies from which they were obtained; the only exception is the Kirby dataset, which had been aligned using Kallisto and whose expression values are calculated using the logarithm of the transcripts per kilobase million (TPM).

Across all datasets, we used GEO platform descriptions as the primary source of probe and gene annotations when available, otherwise original annotations as published by the authors were used for non-standard gene expression profiling platforms. The full set of gene annotation platforms across all expression sets can be found in the metadata files associated with each Bioconductor package, and is additionally provided in Supplementary Tables S11–S13. Gene symbols and Entrez Gene identifiers that matched the probeset ids of a given expression set were subsequently saved as part of the featureData (fData) pertaining to that expression set. For genes with multiple probesets, the *iqr* function within R was used to calculate the variance of the probes across the dataset; only the probe with the highest variance across the dataset was used to calculate the prognostic value of the gene. Standardization of gene expression values (normalization) across datasets was undergone using a meta-analysis (each gene is evaluated in each dataset, and a final estimate was determined for each gene via the survcomp *comb*.*est* function. Further details are provided below).

### MetaGxData package implementation

The breast, ovarian, and pancreatic cancer datasets are available through the MetaGxBreast, MetaGxOvarian, and MetaGxPancreas R data packages hosted on Bioconductor’s ExperimentHub. The MetaGxData packages allow users to select and filter the finalized curated datasets using the loadOvarianDatasets, loadBreastDatasets and loadPancreasDatasets functions of MetaGxOvarian, MetaGxBreast and MetaGxPancreas, respectively. Users are provided options for filtering samples based on clinical parameters, availability of survival data, and sample replicates (patients with highly correlated transcriptomic profiles; spearman correlation > 0.98). Users are also provided other options including, but not limited to, the ability to remove datasets based on the number of samples and the number of survival events present in the data. Importantly, users have the ability to specifically select for only primary tumour samples or several tissue types (primary tumours, healthy tissue, etc.) using the sample type info found in the clinical data.

Collectively, our data compendium, referred to as *MetaGxData*, encompasses 86 processed datasets, containing in total 15,249 breast, ovarian and pancreas samples. Information pertaining to the platform, number of samples, number of probes, and number of unique genes present in the breast, ovarian, and pancreas datasets can be found in in the supplementary files (Supplementary Tables S11, S12 and S13). Expression datasets are represented as SummarizedExperiment objects with attached clinical data (pData), and feature data (fData) and can be loaded into R with a single function call allowing for fast and flexible analysis^38^. Hosting the datasets within the Bioconductor ExperimentHub facilitates rapid integration of new datasets into the existing framework and allows for easy extension of newer studies into the package in future iterations of *MetaGxData*.

### Prognostication of breast and ovarian cancer genes and signature generation

Cox proportional hazards analysis was performed using the R package *survcomp* (version 1.29.4) to estimate the prognostic value (hazard ratio) and significance (corresponding p-value) of the genes in each dataset^64^. In these analyses, overall survival was used as the primary endpoint when determining the hazard ratio. After determining the hazard ratio in each dataset, a final combined estimate of the hazard ratio was calculated using a random-effects model (combine.est from *survcomp*)^65^. Expression data from non-tumor samples was removed from all analyses. When stratifying samples into groups to generate survival curves, samples within each dataset were stratified into two groups based on the median expression of the gene or the median gene signature/module score for all the samples within that dataset. For the gene signatures, risk prediction scores were determined using the signed average of the patients’ gene expression, with the sign being determined as their direction of association with the survival outcome (HR > 1 positive direction). Datasets which did not include the 3 genes in our SCM gene subtype classification model were removed from the survival analyses. For example, the UNC4 breast cancer dataset was excluded, as the ER probe was deemed poor quality by the manufacturer and removed from the annotations. Furthermore, the ICGCSEQ dataset in MetaGxPancreas was excluded, due to overlap of a subset of patients with the ICGCMICRO dataset. To generate the MetaGx gene signature, the aforementioned analysis was performed on common genes in MetaGxBreast and MetaGxOvarian to determine the hazard ratios of each gene. The METABRIC dataset (n = 2136 samples) from MetaGxBreast, and 5 of the largest ovarian datasets (GSE9891, GSE32062, GSE49997, GSE26712, GSE51088, totaling 1,116 samples) were removed from the analysis for later use as the validation cohort to test the signature. The 53 genes with significant hazard ratios in both cancers (FDR < 5% and HR > 1.125 or FDR < 5% and HR < 0.875) were selected for the MetaGx gene signature.

### Correlation between the signature scores and gene modules

Correlations between the MetaGx signature and the gene modules were determined by finding the individual Spearman correlations coefficients between the signatures risk predictions, and the gene modules risk predictions in each individual dataset. A meta-estimate for the correlation coefficient was then determined from the individual correlation coefficients and their associated standard errors via the survcomp package (combine.est function) using a random effects model.

### Statistical analysis

The hazard ratios were computed via the R survcomp package as D indices by using risk predictions for the signatures along with the patients’ corresponding survival times and overall survival statuses. The D-index is a robust estimate of the traditional Cox’s hazard ratio, more precisely an estimate of the hazard ratio comparing two equal-sized prognostic groups^64,66^. This is a scale-free measure of separation between two independent survival distributions under the proportional hazards assumption. All individual estimates were combined into a meta-estimate via *survcomp* in a random effects model to obtain a single best estimate of the D index; this metric is reported throughout the present work. The patient groups, survival times and overall survival status of the patients from all the datasets were used within the survival package to generate Kaplan-Meir survival curves and determine the log-rank test p values^67^. D index and log-rank test p values below 0.05 were considered to be statistically significant. All analyses were conducted using R.

## Supplementary information

Supplementary Information

Supplementary File S6

## Data Availability

The datasets used in this manuscript are all publicly available for download through R Bioconductor’s ExperimentHub (https://bioconductor.org/packages/release/data/experiment/). The breast, ovarian, and pancreas datasets can be found in MetaGxbreast, MetaGxOvarian, and MetaGxPancreas, respectively. All the code required to reproduce the single-gene prognosis analysis, as well as the genome-wide meta-analysis and signature results, is publicly available on the CodeOcean (https://codeocean.com, analysis at https://codeocean.com/capsule/6438633/). The CodeOcean contains an executable version of the code, in the form of a standalone docker, that can be used to generate all of the results in the present work. This work complies with the guidelines outlined in^68–70^.
