# Supplementary material for: MetaGxData: Clinically Annotated Breast, Ovarian and Pancreatic Cancer Datasets and their Use in Generating a Multi-Cancer Gene Signature
Source: Sci Rep. 2019 Jun 19;9:8770. doi: 10.1038/s41598-019-45165-4 (PMC6584731; doi:10.1038/s41598-019-45165-4)
Supplement: Supplementary file 1 — Supplementary Information [file 41598_2019_45165_MOESM1_ESM.pdf]

SUPPLEMENTARY INFORMATION

MetaGxData: Clinically Annotated Breast, Ovarian and Pancreatic Cancer Datasets and their Use in Generating a Multi-Cancer Gene Signature

Deena M.A. Gendoo [1+*], Michael Zon[2,4+], Vandana Sandhu [2], Venkata SK Manem [2,3,5], Natchar Ratanasirigulchai [2], Gregory M Chen[2], Levi Waldron [6 *‡], Benjamin Haibe-Kains [2,3,7,8,9*‡]

[1] Centre for Computational Biology, Institute of Cancer and Genomic Sciences, University of Birmingham, Birmingham, United Kingdom
[2] Princess Margaret Cancer Center, University Health Network, Toronto, M5G 2C1, Canada
[3] Department of Medical Biophysics, University of Toronto, Toronto, M5S 3H7, Canada
[4] Department of Biomedical Engineering, McMaster University, Toronto, L8S 4L8, Canada
[5] Institut Universitaire de Cardiologie et de Pneumologie de Québec, Université Laval, G1V 4G5, Canada
[6] Graduate School of Public Health and Health Policy, Institute of Implementation Science in Population Health, City University of New York School, New York, 11101, USA
[7] Department of Computer Science, University of Toronto, Toronto, M5T 3A1, Canada
[8] Ontario Institute of Cancer Research, Toronto, M5G 0A3, Canada
[9] Vector Institute, Toronto, M5G 1M1, Canada

[+] These authors contributed equally to this work
[‡] Co-senior authors

* Corresponding authors
* Correspondence to: d.gendoo@bham.ac.uk [DMAG], levi.waldron@hunter.cuny.edu [LW], and benjamin.haibe.kains@utoronto.ca [BHK]

Supplementary Information

Supplementary File S1. Explanation of curated clinical annotations (phenotype data variables) in MetaGxBreast (A), MetaGxOvarian (B), and MetaGxPancreas (C).

Supplementary Figure S2. Heatmap representation of clinical variables availability across gene expression datasets of MetaGxBreast, MetaGxOvarian, and MetaGxPancreas. Datasets are represented as rows and clinical variables as columns. The percentage of samples in each dataset that is annotated with a particular variable is represented.

Supplementary Figure S3. Schematic representation of the clinical variables (pData) that are available across gene expression datasets in MetaGxBreast. The Stacked bar plots indicate the percentage of samples in every dataset annotated with a particular variable designation. Continuous numeric values are represented by box plots.

Supplementary Figure S4. Schematic representation of the clinical variables (pData) that are available across gene expression datasets in MetaGxOvarian. The Stacked bar plots indicate the percentage of samples in every dataset annotated with a particular variable designation. Continuous numeric values are represented by box plots.

Supplementary Figure S5. Schematic representation of the clinical variables (pData) that are available across gene expression datasets in MetaGxPancreas. The Stacked bar plots indicate the percentage of samples in every dataset annotated with a particular variable designation. Continuous numeric values are represented by box plots.

Supplementary File S6. (A) A Venn diagram of significant genes (FDR<5%) in breast and ovarian cancers, following calculation of the Hazards Ratio is indicated (top). A total of 695 and 654 significantly prognostic genes were identified for ovarian and breast cancer, respectively. Common significant genes between both tumour types (n=30) were further subdivided by their log hazard ratio, for each tumour type. Genes for which elevated expression levels are prognostic (HR>1) across both tumours, or genes for which down-regulated expression is prognostic (HR<1) are indicated. **(B)** A list of the computed Hazard Ratio of all genes, using MetaGxBreast, MetaGxOvarian, and MetaGxPancreas is provided.

Supplementary Figure S7. Survival curves for the metaGx, mammaprint signature, and oncotype signature in the ER-/HER2- breast cancer patients stratified by molecular subtypes. The asterisks above the D indices indicate whether the D index was statistically significant ($p < 0.05$).

Supplementary File S8. Information pertaining to the genes comprising each of the pancreatic signatures tested against the MetaGx signature. Additional information is also provided in Table2.

Supplementary Figure S9. Correlation between MetaGx signature risk prediction scores and their gene module scores.

Supplementary Figure S10. Density plots comparing the prognostic value of the gene signature in breast, ovarian, and pancreatic cancer to 1000 random signatures of the same length. The dashed line represents the location of the D index of the signature.

Supplementary Table S11. Breast cancer dataset information. The ’patients’ column indicates the total number of subjects in each study.

Supplementary Table S12. Ovarian cancer dataset information. The ’patients’ column indicates the total number of subjects in each study.

Supplementary Table S13. Pancreatic cancer dataset information. The ’patients’ column indicates the total number of subjects in each study.

Supplementary Figures

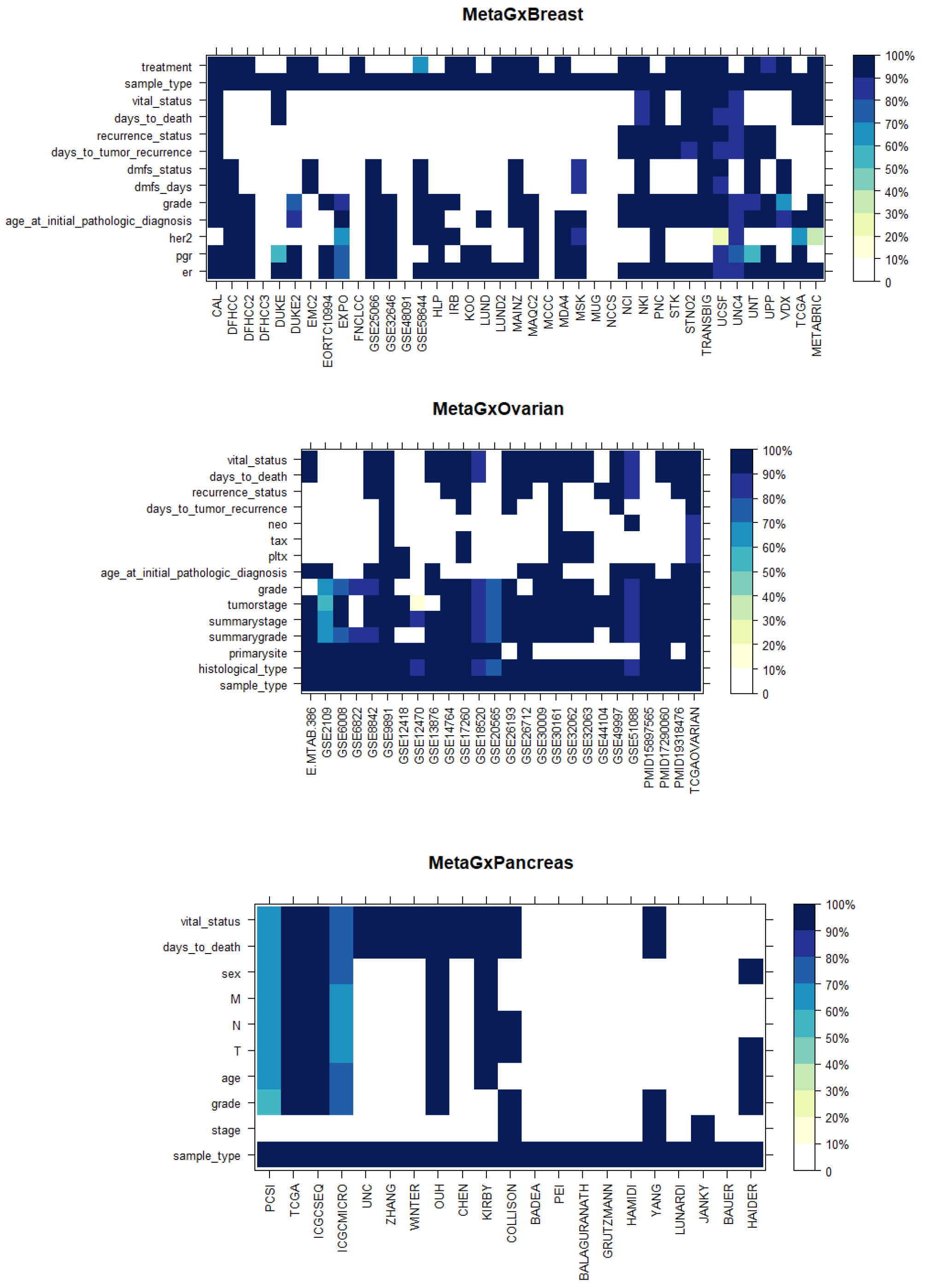

Supplementary Figure S2: Heatmap representation of clinical variables availability across gene expression datasets of MetaGxBreast, MetaGxOvarian, and MetaGxPancreas. Datasets are represented as rows and clinical variables as columns. The percentage of samples in each dataset that is annotated with a particular variable is represented.

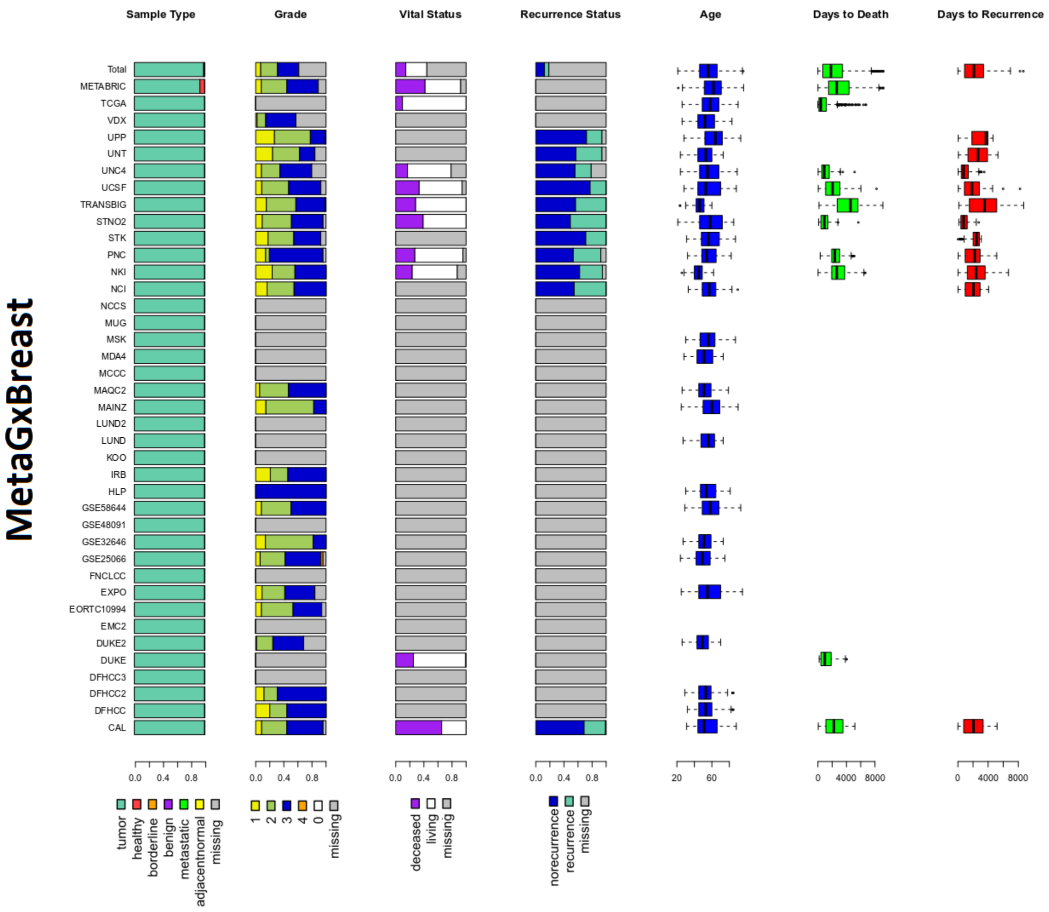

Supplementary Figure S3: Schematic representation of the clinical variables (pData) that are available across gene expression datasets in MetaGxBreast. The Stacked bar plots indicate the percentage of samples in every dataset annotated with a particular variable designation. Continuous numeric values are represented by box plots.

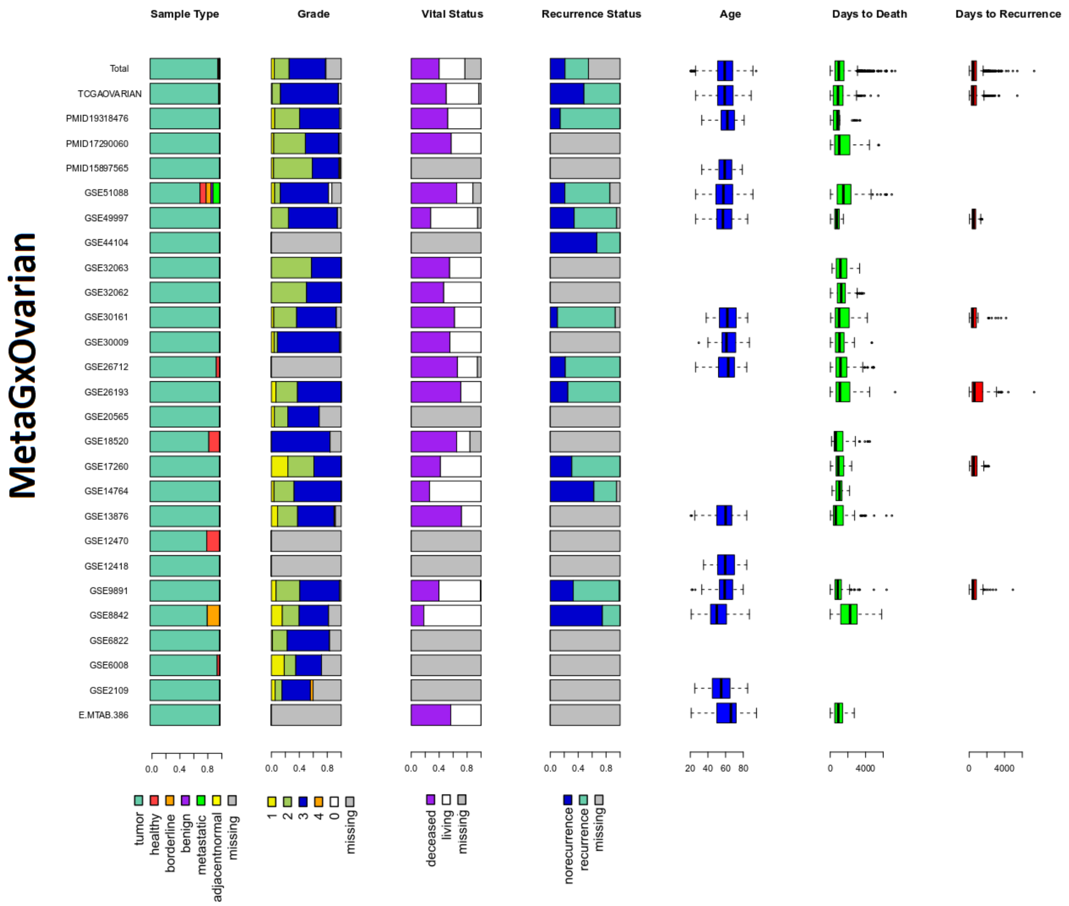

Supplementary Figure S4: Schematic representation of the clinical variables (pData) that are available across gene expression datasets in MetaGxOvarian. The Stacked bar plots indicate the percentage of samples in every dataset annotated with a particular variable designation. Continuous numeric values are represented by box plots.

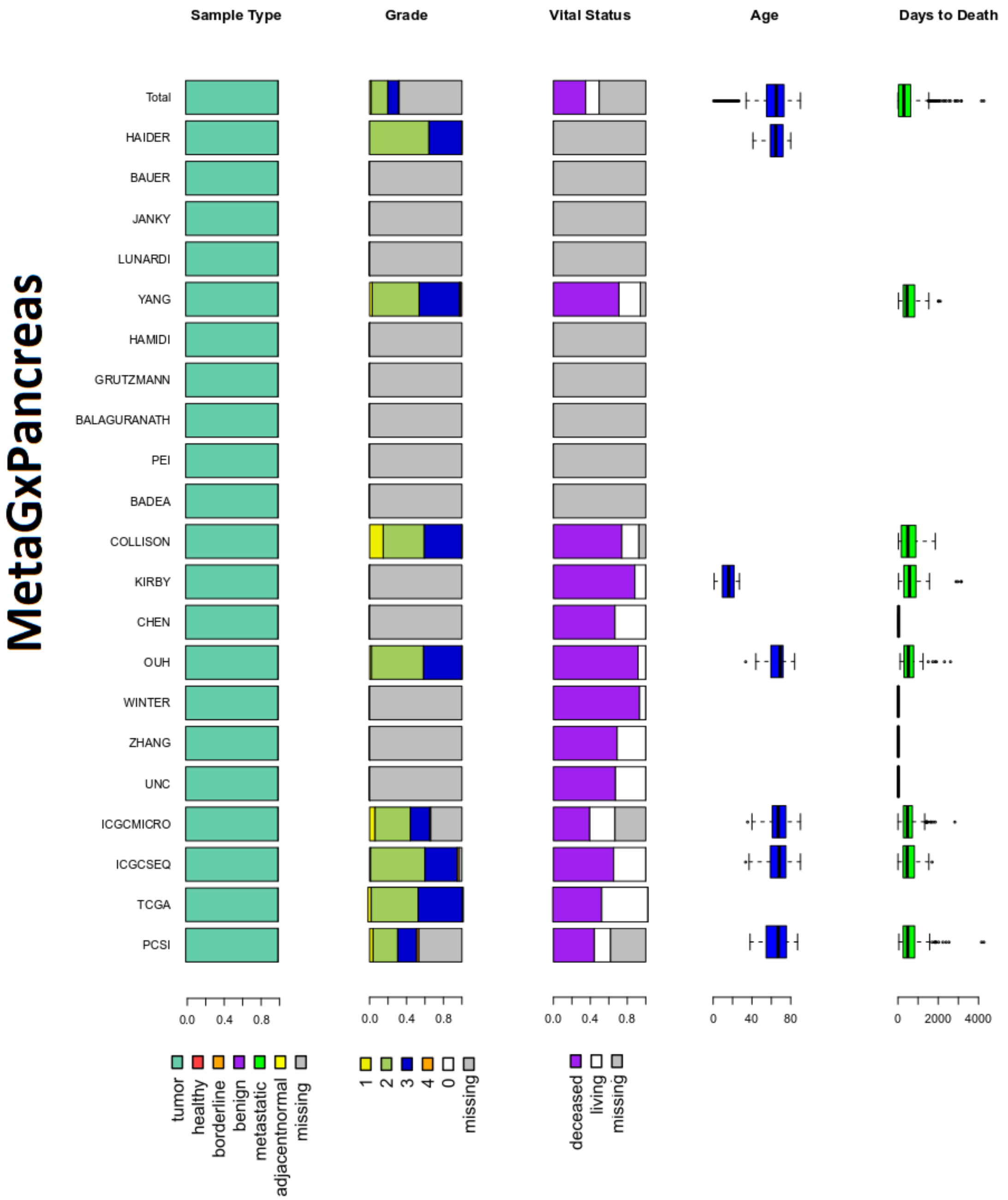

Supplementary Figure S5: Schematic representation of the clinical variables (pData) that are available across gene expression datasets in MetaGxPancreas. The Stacked bar plots indicate the percentage of samples in every dataset annotated with a particular variable designation. Continuous numeric values are represented by box plots.

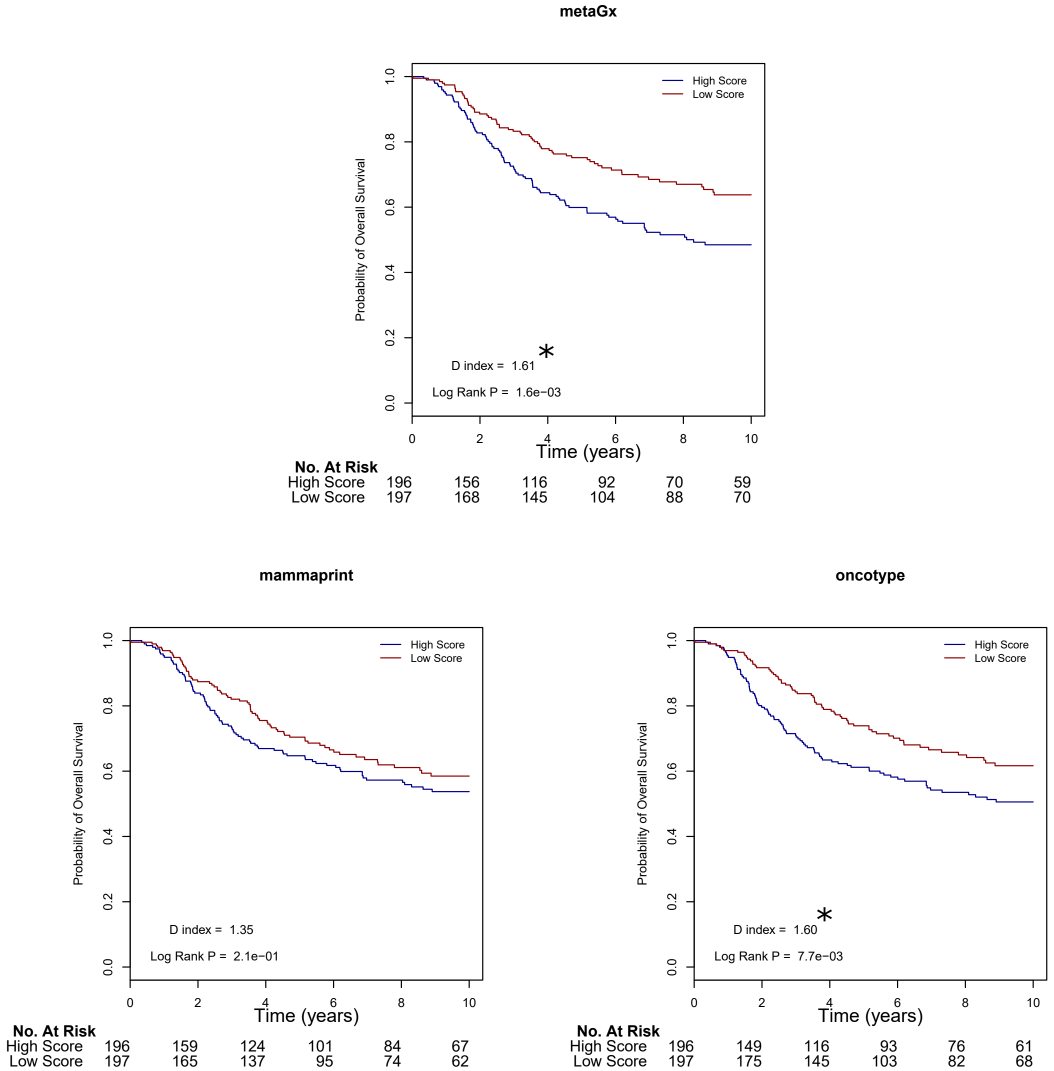

Supplementary Figure S7: Survival curves for the metaGx, mammaprint signature, and oncotype signature in the ER-/HER2- breast cancer patients stratified by molecular subtypes. The asterisks above the D indices indicate whether the D index was statistically significant ($p < 0.05$).

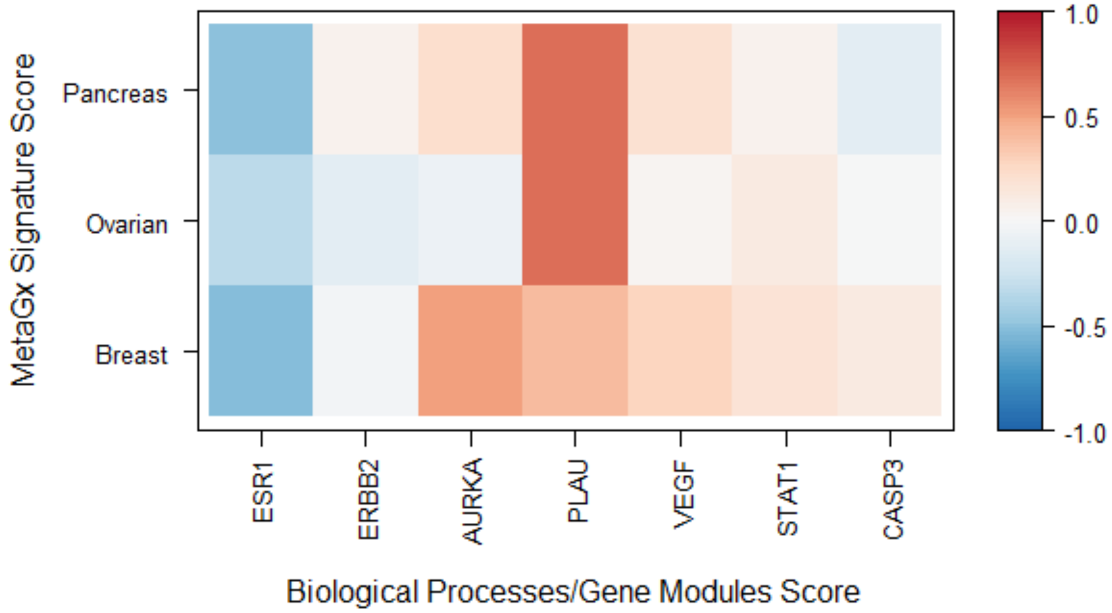

Supplementary Figure S9: Correlation between the patients MetaGx signature risk prediction scores and their gene module scores.

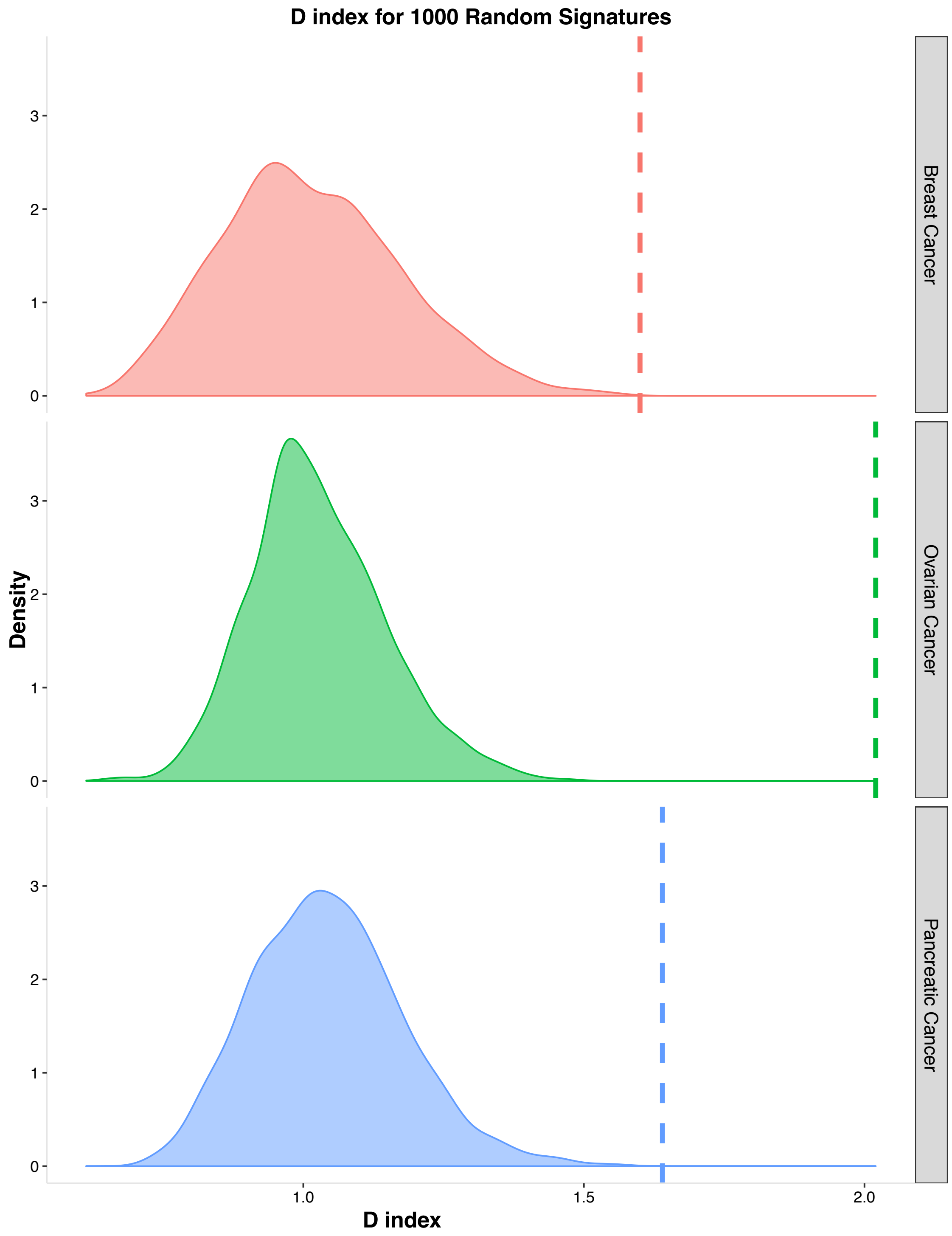

Supplementary Figure S10: Density plots comparing the prognostic value of the gene signature in breast, ovarian, and pancreatic cancer to 1000 random signatures of the same length. The dashed line represents the location of the D index of the signature.

Supplementary Tables

	Dataset	PMID	Accession	Platform	Sample Prep	Patients	Probes	Genes
1	CAL	17157792	E-TABM-158	Affymetrix HGU	FF	118	21169	13266
2	DFHCC	20098429	GSE19615	GPL570	FF	115	42447	21024
3	DFHCC2	20100965	GSE18864	GPL570	FF	84	42447	21024
4	DFHCC3	16473279	GSE3744	GPL570	FF	40	42447	21024
5	DUKE	16273092	GSE3143	GPL8300	FF	171	12085	9103
6	DUKE2	18024211	GSE6861	GPL1352	FF	160	45490	20989
7	EMC2	19421193	GSE12276	GPL570	FF	204	42447	21024
8	EORTC 10994	15897907	GSE1561	GPL96	FF	49	20967	13209
9	EXPO	NA	GSE2109	GPL570	NA	353	42447	21024
10	FNCLCC	17659439	GSE7017	GPL4819	FF	150	6064	5109
11	HLP	19688261	E-TABM-543	Illumina	FF	53	26536	19451
12	IRB	18297396	GSE5460	GPL570	FF	129	42447	21024
13	KOO	12747878	Authors' website	Affymetrix HGU95	NA	88	280	258
14	LUND	18430221	GSE31863	GPL14374	FF	143	11154	10513
15	LUND2	17452630	GSE5325	GPL3883	FF	105	22008	12676
16	MAINZ	18593943	GSE11121	GPL96	FF	200	20967	13209
17	MAQC2	20064235	GSE20194	GPL96///GPL570///GPL1261	NA	230	20967	13209
18	MCCC	19960244	GSE19177	GPL6106	FF	75	19048	14959
19	MDA4	16896004	MDACC DB	Affymetrix HGU	NA	129	21169	13266
20	MSK	16049480	GSE2603	GPL96	FF	99	20967	13209
21	MUG	18592372	GSE10510	GPL6486	FF	152	14288	10721
22	NCCS	18636107	GSE5364	GPL96	FF	183	20967	13209
23	NCI	12917485	Authors' website	In-house cDNA	NA	99	5154	4112
24	NKI	12490681	Rosetta Inpharmatics	Agilent	FF	337	14960	13120
25	PNC	21910250	GSE20711	GPL570	FF	92	42447	21024
26	STK	16280042	GSE1456	GPL97///GPL96	FF	159	36178	19092
27	STNO2	12829800	GSE4382	GPL180///GPL2776///GPL2777	FF	118	3663	3230
28	TRANSBIG	17545524	GSE7390	GPL96	FF	198	20967	13209
29	UCSF	17428335	Authors' website	In-house cDNA	FF	162	8015	6279
30	UNC4	20813035	GSE18229	GPL885///GPL887///GPL1390	FF	305	5420	5026
31	UNT	16478745	GSE2990	Affymetrix HGU	FF	133	36084	18821
32	UPP	16141321	GSE3494	GPL570	FF	251	36178	19092
33	VDX	17420468	GSE2034	Affymetrix HGU	FF	344	21169	13266
34	METABRIC	22522925	EGAS0000 0000083	METABRIC	FF	2136	36155	24924
35	TCGA	23000897	TCGA Website	TCGA	FF	1073	19504	19408
36	GSE25066	21558518	GSE25066	GPL96	FF	508	20967	13209
37	GSE32646	22320227	GSE32646	GPL570	FF	115	42437	21024
38	GSE58644	25284793	GSE58644	GPL6244	FF	321	21462	20202
39	GSE48091	26077471	GSE48091	GPL10379	FF	623	23246	12917

Supplementary Table S11: Breast cancer dataset information. The 'patients' column indicates the total number of subjects in each study.

	Dataset	PMID	Accession	Platform	Sample Prep	Patients	Probes	Genes
1	E.MTAB.386	22348002	E.MTAB.386	GPL6104	FFPE	129	12449	10572
2	GSE12418	16996261	GSE12418	GPL5886	FF	54	11304	10559
3	GSE12470	19486012	GSE12470	GPL887	FF	53	15999	13717
4	GSE13876	19192944	GSE13876	GPL7759	FF	157	20939	13894
5	GSE14764	19294737	GSE14764	GPL96	FF	80	20967	13209
6	GSE17260	20300634	GSE17260	GPL6480	FF	110	30936	19596
7	GSE18520	19962670	GSE18520	GPL570	FF	63	42447	21024
8	GSE20565	20492709	GSE20565	GPL570///GPL2005///GPL6801	FF	140	42447	21024
9	GSE2109	NA	GSE2109	GPL570	NA	204	42447	21024
10	GSE26193	22101765	GSE26193	GPL570	FF	107	42447	21024
11	GSE26712	18593951	GSE26712	GPL96	FF	195	20967	13209
12	GSE30009	22492981	GSE30009	GPL13728	FF	103	363	363
13	GSE30161	22348014	GSE30161	GPL570	FFPE	58	42447	21024
14	GSE32062	22241791	GSE32062	GPL570///GPL6480	FF	260	30936	19596
15	GSE32063	22241791	GSE32063	GPL6480	FF	40	30936	19596
16	GSE44104	23934190	GSE44104	GPL570	FF	60	42447	21024
17	GSE49997	22497737	GSE49997	GPL2986	FF	204	18439	16760
18	GSE51088	24368280	GSE51088	GPL7264	FF	172	18703	16747
19	GSE6008	17418409	GSE6008	GPL96	FF	103	20967	13209
20	GSE6822	NA	GSE6822	GPL80	NA	66	6407	5581
21	GSE8842	19047114	GSE8842	GPL5689	FF	83	7809	6250
22	GSE9891	18698038	GSE9891	GPL570	FF	285	42447	21024
23	PMID15897565	15897565	PMID15897565	GPL96	FF	63	20967	13209
24	PMID17290060	17290060	PMID17290060	GPL96	FF	117	20967	13209
25	PMID19318476	19318476	PMID19318476	GPL96	FF	42	20967	13209
26	TCGAOVARIAN	NA	TCGA	GPL4685	FF	578	21260	13224

Supplementary Table S12: Ovarian cancer dataset information. The 'patients' column indicates the total number of subjects in each study.

	Dataset	PMID	Accession	Platform	Sample Prep	Patients	Probes	Genes
1	BADEA	19260470	GSE15471	Affymetrix Human Genome U133 Plus 2.0 Array	FF	39	20590	20316
2	BALAGURANATH	18765825	GSE11838	Human 1A Microarray G4110A-G4110B	FFPE	28	15254	15115
3	CHEN	26247463	GSE57495	Affymetrix,Rosetta-Merck RSTA Custom 2.0	FF	63	22115	18207
4	COLLISON	21460848	GSE17891	Affymetrix, array U133 Plus 2.0	FFPE	27	22408	21711
5	GRUTZMANN	15548371	E.MEXP.950	Affymetrix GeneChip Human Genome HG-U133B	FF	11	19926	19265
6	ICGCSEQ	21436628	icgc.org	Illumina HumanHT-12 V4.0 expression beadchip	FF	95	15965	15602
7	ICGCMICRO	21436628	icgc.org	Illumina HiSeq 2000-2500	FF	267	21701	21375
8	KIRBY	27282075	GSE79670	Illumina, RNA seqencing HiSeq	FF	51	26264	25276
9	OUH	25579086	GSE60980	Agilent-028004 SurePrint G3 Human GE 8x60K Microarray	FF	48	32664	17991
10	PCSI	NA	NA	Illumina HiSeq 2000-2500	FF	210	58682	34941
11	PEI	19732725	GSE16515	Affymetrix Human Genome U133 Plus 2.0 Array	FF	36	22434	21353
12	TCGA	NA	TCGA-PAAD	IlluminaHiSeq_RNASeqV2	FF	146	20501	17948
13	UNC	26343385	GSE71729	Agilent-014850 Whole Human Genome Microarray 4x44K G4112F Agilent-014850 Whole Human Genome Microarray 4x44K G4112F	FF	125	19751	16074
14	WINTER	22615549	E.MEXP.2780	A-AFFY-44-Affymetrix GeneChip Human Genome U133 Plus 2.0 [HG-U133_Plus_2]	FF	30	22408	21711
15	ZHANG	22363658	GSE28735	Affymetrix GeneChip Human Gene 1.0 ST arrays	FF	42	24215	23220
16	HAMIDI	NA	GSE77858	Agilent-012097 Human 1A Microarray (V2) G4110B	FF	46	16898	16898
17	YANG	27197190	GSE62452	Affymetrix GeneChip Human Gene 1.0 ST		69	23306	22213
18	LUNARDI	25415223	GSE55643	Agilent-014850 Whole Human Genome Microarray 4x44K G4112F	FF	45	19595	17231
19	JANKY	27520560	GSE62165	Affymetrix Human Genome U219 Array	FF	118	20395	20395
20	BAUER	27578530	E-MTAB-1791	Illumina human WG6 Expression BeadChip		195	19936	19936
21	HAIDER	28592875	GSE56560	Affymetrix Human Exon 1.0 ST Array	FF	28	17803	15999

Supplementary Table S13: Pancreas cancer dataset information. The 'patients' column indicates the total number of subjects in each study.

Supplementary File S1

Curated Clinical Annotations in MetaGxBreast

col.name	var.class	uniqueness	requiredness	allowedvalues	description
sample_name	character	unique	required	*	primary sample identifier
alt_sample_name	character	unique	optional	*	if another identifier is used, for example in supplemental tables
unique_patient_ID	character	non-unique	optional	*	Use this column if there are technical replicates. If this column contains non-unique entries, expression values of those arrays will eventually be averaged.
sample_type	character	non-unique	optional	tumor|metastatic|healthy|adjacentnormal	healthy should be only from individuals without cancer, metastatic for non-primary tumors
er	character	non-unique	optional	positive|negative	Estrogen Receptor status
pgr	character	non-unique	optional	positive|negative	Progesterone Receptor status
her2	character	non-unique	optional	positive|negative	HER2 status
tumor_size	decimalonly	non-unique	optional	decimal	tumor size in centimeters
T	integer	non-unique	optional	1|2|3|4	T (1-4): size in TNM staging: see http://www.cancer.gov/cancertopics/pdq/treatment/breast/healthprofessional/page3.
N	integer	non-unique	optional	0|1	N (0/1): spread to regional lymph nodes no/yes
age_at_initial_pathologic_diagnosis	numeric	non-unique	optional	decimal	age at initial pathologic diagnosis
grade	integer	non-unique	optional	1|2|3	Taken directly from Haibe-Kains paper, PMID 22262870

dmfs_days	numeric	non-unique	optional	decimal	Potentially censored number of days from diagnosis to distant metastasis or death (dmfs).
dmfs_status	character	non-unique	optional	norecurrence|recurrence	Distant metastasis-free survival (dmfs) censoring variable.
days_to_tumor_recurrence	integer	non-unique	optional	decimal	This is a potentially censored time from diagnosis to recurrence (rfs).
recurrence_status	character	non-unique	optional	norecurrence|recurrence	recurrence (rfs) status censoring variable
days_to_death	integer	non-unique	optional	decimal	This is a potentially censored time from diagnosis to death.
vital_status	character	non-unique	optional	living|deceased	overall survival censoring variable
tissue	numeric	non-unique	optional	normal|tumor|DCIS|metastasis.or.recurrence|after.treatment	
treatment	integer	non-unique	optional	untreated|chemotherapy|hormonotherapy|endocrine|chemo.plus.hormono	based on observing whether tamoxifen, herceptin, and chemotherapy were administered to a patient
percent_normal_cells	character	non-unique	optional	\d+[+-]?	estimated percentage of normal cells
percent_stromal_cells	character	non-unique	optional	\d+[+-]?	estimated percentage of stromal cells
percent_tumor_cells	character	non-unique	optional	\d+[+-]?	estimated percentage of tumor cells
batch	character	non-unique	optional	*	batch variable - hybridization date when Affymetrix CEL files are available.

uncurated_author_meta data	character	non-unique	optional	*	original uncurated data, with each field separated by ///.
duplicates	character	non-unique	optional	*	list of sample replicates across all expression sets

Curated Clinical Annotations in MetaGxOvarian

col.name	var.class	uniqueness	requiredness	allowedvalues	description
sample_name	character	unique	required	*	primary sample identifier
alt_sample_name	character	unique	optional	*	if another identifier is used, for example in supplemental tables
unique_patient_ID	character	non-unique	optional	*	Use this column if there are technical replicates. If this column contains non-unique entries, expression values of those arrays will eventually be averaged.
sample_type	character	non-unique	required	tumor|metastatic|borderline|benign|adjacentnormal|healthy|cellline	healthy should be only from individuals without cancer, adjacentnormal from individuals with cancer, metastatic for non-primary tumors, borderline includes both borderline and LMP tumors, benign for benign tumors.
histological_type	character	non-unique	optional	ser|endo|clearcell|mucinous|undifferentiated|other|mix	ser=serous;endo=endometrioid;clearcell=mixture of ser+endo. Other includes sarcomatoid, endometroid, papillary serous, unspecified adenocarcinoma, dysgerminoma
primarysite	character	non-unique	optional	ov|ft|other	ov=ovary;ft=fallopian tube
arrayedsite	character	non-unique	optional	ov|ft|other	ov=ovary;"ft=fallopian tube"
summarygrade	character	non-unique	optional	low|high	low = 1, 2, LMP. High= 3,4,23
summarystage	character	non-unique	optional	early|late	early = 1,2, 12. late=3,4,23,34

tumorstage	character	non-unique	optional	1|2|3|4	Group Stage (I-IV, translated to 1-4 for R usage.). If multiple stages given (eg 34), use the highest.
substage	character	non-unique	optional	a|b|c|d	substage (abcd). For cases like ab, bc, use highest given
grade	integer	non-unique	optional	0|1|2|3|4	G (0-4): If multiple given, ie 12, 23, use highest given
age_at_initial_pathologic_diagnosis	integer	non-unique	optional	[1-9][0-9]	in years
pltx	character	non-unique	optional	[yn]	patient treated with platin
tax	character	non-unique	optional	[yn]	patient treated with taxol
neo	character	non-unique	optional	[yn]	neoadjuvant treatment
days_to_tumor_recurrence	numeric	non-unique	optional	decimal	time to recurrence or last follow-up in days
recurrence_status	character	non-unique	optional	recurrence|norecurrence	recurrence censoring variable
days_to_death	numeric	non-unique	optional	decimal	time to death or last follow-up in days
vital_status	character	non-unique	optional	living|deceased	Overall survival censoring variable
os_binary	character	non-unique	optional	short|long	dichotomized overall survival variable (short, long)
relapse_binary	character	non-unique	optional	short|long	dichotomized relapse variable (short, long)
site_of_tumor_first_recurrence	character	non-unique	optional	metastasis|locoregional|none|locoregional_plus_metastatic	none for no recurrence, na for unknown
primary_therapy_outcome_success	character	non-unique	optional	completeresponse|partialresponse|progressive	response to any kind of therapy

				disease|stabledisease	
debulking	character	non-unique	optional	optimal|suboptimal	amount of residual disease (optimal = <1cm, suboptimal=>1cm)
percent_normal_cells	character	non-unique	optional	\d+[+-]?	estimated percentage of normal cells
percent_stromal_cells	character	non-unique	optional	\d+[+-]?	estimated percentage of stromal cells
percent_tumor_cells	character	non-unique	optional	\d+[+-]?	estimated percentage of tumor cells
batch	character	non-unique	optional	*	batch variable - hybridization date when Affymetrix CEL files are available
flag	character	non-unique	optional	*	Free-form flag to annotate the sample.
flag_notes	character	non-unique	optional	*	Notes associated with the flag.
uncurated_author_metadata	character	non-unique	optional	*	original uncurated data, with each field separated by ///.
duplicates	character	non-unique	optional	*	list of sample replicates across all expression sets

Curated Clinical Annotations in MetaGxPancreas

**col.name**	**var.class**	**uniqueness**	**requiredness**	**allowedvalues**	**description**
sample_name	character	unique	required	*	primary sample identifier
T	integer	non-unique	optional	1|2|3|4	T (1-4): size in TNM staging: see http://www.cancer.gov/cancertopics/pdq/treatment/breast/healthprofessional/page3.
N	integer	non-unique	optional	Nx |N0}N1|N2|N3	Standard N system used in TNM, i.e Nx (cannot be assessed) | N0 no regional lymph node metastasis | N1

					metastasis present | N2 tumor spread to extent between N1 and N3 | N3 tumor spread to distant lymph nodes
grade	integer	non-unique	optional	G1|G2|G3	G1 low grade | G2 intermediate grade | G3 high grade
days_to_death	integer	non-unique	optional	decimal	This is a potentially censored time from diagnosis to death.
vital_status	character	non-unique	optional	living|deceased	overall survival censoring variable
unique_patient_id	character	unique	optional	*	additional sample identification info from the dataset
sex	character	non-unique	optional	male|femal	The patient's sex
M	character	non-unique	optional	Mx|M0|M1	distant metastases info, Mx is unknown, M0 implies cancer has not spread and M1 implies cancer has spread
sample_type	character	non-unique	required	tumour	All samples were from primary PDAC tumors
geo_accession	character	non-unique	optional	*	patient's geo accession number
pTNM	character	non-unique	optional	R0|R1	Resected margin status, R0 no cancer cells at margin, R1 cancer cells at marging
cellularity	integer	non-unique	optional	0-100	percent cellularity
age	integer	non-unique	optional	[1-9][0-9]	The patient's age in years
age at Dx (surgery)	integer	non-unique	optional	[1-9][0-9]	The patient's age in years at the time of surgery

Supplementary File S8

MetaGx Signature

	Symbol	Direction	Entrez ID	Ensemble ID	Description
1	CRABP2	1	1382	ENSG00000143320	cellular retinoic acid binding protein 2
2	DPP4	1	1803	ENSG00000197635	dipeptidyl peptidase 4
3	EGFR	1	1956	ENSG00000146648	epidermal growth factor receptor
4	LOXL2	1	4017	ENSG00000134013	lysyl oxidase like 2
5	OPRM1	1	4988	ENSG00000112038	opioid receptor mu 1
6	SERPINB2	1	5055	ENSG00000197632	serpin family B member 2
7	PLAU	1	5328	ENSG00000122861	plasminogen activator, urokinase
8	PPEF1	1	5475	ENSG00000086717	protein phosphatase with EF-hand domain 1
9	STK3	1	6788	ENSG00000104375	serine/threonine kinase 3
10	NAE1	1	8883	ENSG00000159593	NEDD8 activating enzyme E1 subunit 1
11	ACTN4	1	81	ENSG00000130402	actinin alpha 4
12	CACNB3	1	784	ENSG00000167535	calcium voltage-gated channel auxiliary subunit beta 3
13	CBFB	1	865	ENSG00000067955	core-binding factor beta subunit
14	IMPDH1	1	3614	ENSG00000106348	inosine monophosphate dehydrogenase 1
15	IRF3	1	3661	ENSG00000126456	interferon regulatory factor 3
16	KIF11	1	3832	ENSG00000138160	kinesin family member 11
17	TERF2	1	7014	ENSG00000132604	telomeric repeat binding factor 2
18	TGFBI	1	7045	ENSG00000120708	transforming growth factor beta induced
19	TPD52L2	1	7165	ENSG00000101150	tumor protein D52 like 2
20	MSC	1	9242	ENSG00000178860	musculin
21	PWP1	1	11137	ENSG00000136045	PWP1 homolog, endonuclein
22	CLDN4	1	1364	ENSG00000189143	claudin 4
23	CSE1L	1	1434	ENSG00000124207	chromosome segregation 1 like

24	NID2	1	22795	ENSG00000087303	nidogen 2
25	RALY	1	22913	ENSG00000125970	RALY heterogeneous nuclear ribonucleoprotein
26	CDK19	1	23097	ENSG00000155111	cyclin dependent kinase 19
27	ACKR3	1	57007	ENSG00000144476	atypical chemokine receptor 3
28	TEX261	1	113419	ENSG00000144043	testis expressed 261
29	LATS2	1	26524	ENSG00000150457	large tumor suppressor kinase 2
30	REX1BD	1	55049	ENSG00000006015	required for excision 1-B domain containing
31	DARS2	1	55157	ENSG00000117593	aspartyl-tRNA synthetase 2, mitochondrial
32	UTP6	1	55813	ENSG00000108651	UTP6, small subunit processome component
33	CAMK1D	1	57118	ENSG00000183049	calcium/calmodulin dependent protein kinase ID
34	SIPA1L2	1	57568	ENSG00000116991	signal induced proliferation associated 1 like 2
35	ARHGAP21	1	57584	ENSG00000107863	Rho GTPase activating protein 21
36	COP1	1	64326	ENSG00000143207	COP1, E3 ubiquitin ligase
37	C12orf49	1	79794	ENSG00000111412	chromosome 12 open reading frame 49
38	KATNAL1	1	84056	ENSG00000102781	katanin catalytic subunit A1 like 1
39	ZFAND2A	1	90637	ENSG00000178381	zinc finger AN1-type containing 2A
40	MYADM	1	91663	ENSG00000179820	myeloid associated differentiation marker
41	CMBL	1	134147	ENSG00000164237	carboxymethylenebutenolidase homolog
42	DDB2	-1	1643	ENSG00000134574	damage specific DNA binding protein 2
43	RARRES3	-1	5920	ENSG00000133321	retinoic acid receptor responder 3
44	TNFRSF18	-1	8784	ENSG00000186891	TNF receptor superfamily member 18
45	GSTZ1	-1	2954	ENSG00000100577	glutathione S-transferase zeta 1
46	MOCS1	-1	4337	ENSG00000124615	molybdenum cofactor synthesis 1
47	ZNF204P	-1	7754	#N/A	zinc finger protein 204, pseudogene
48	FAM189A2	-1	9413	ENSG00000135063	family with sequence similarity 189 member A2
49	ZSCAN32	-1	54925	ENSG00000140987	zinc finger and SCAN domain containing 32
50	CDC37L1	-1	55664	ENSG00000106993	cell division cycle 37 like 1
51	MREG	-1	55686	ENSG00000118242	melanoregulin
52	CAMSAP3	-1	57662	ENSG00000076826	calmodulin regulated spectrin associated protein family member 3

53	MYLK3	-1	91807	ENSG00000140795	myosin light chain kinase 3

Plos One 13-Gene Signature

	Symbol	Direction	Entrez ID	Ensemble ID	Description
1	CCDC88C	1	440193	ENSG00000015133	coiled-coil domain containing 88C
2	CD200R1	1	131450	ENSG00000163606	CD200 receptor 1
3	CDH5	1	1003	ENSG00000179776	cadherin 5
4	CUL3	1	8452	ENSG00000036257	cullin 3
5	RIMKLB	1	57494	ENSG00000166532	ribosomal modification protein rimK like family member B
6	ELAVL1	1	1994	ENSG00000066044	ELAV like RNA binding protein 1
7	LINC01341	1	149134	#N/A	long intergenic non-protein coding RNA 1341
8	MDM2	1	4193	ENSG00000135679	MDM2 proto-oncogene
9	MS4A3	1	932	ENSG00000149516	membrane spanning 4-domains A3
10	PLCG1	1	5335	ENSG00000124181	phospholipase C gamma 1
11	PRKCSH	1	5589	ENSG00000130175	protein kinase C substrate 80K-H
12	TGFA	1	7039	ENSG00000163235	transforming growth factor alpha
13	ULBP3	1	79465	ENSG00000131019	UL16 binding protein 3

Genome Medicine Signature

	Symbol	Direction	Entrez ID	Ensemble ID	Description
1	KIF14	1	9928	ENSG00000118193	kinesin family member 14
2	CNNM3	-1	26505	ENSG00000168763	cyclin and CBS domain divalent metal cation transport mediator 3
3	IGF2BP2	1	10644	ENSG00000073792	insulin like growth factor 2 mRNA binding protein 2
4	TMEM26	-1	219623	ENSG00000196932	transmembrane protein 26

5	ARRB1	1	408	ENSG00000137486	arrestin beta 1
6	NPLOC4	1	55666	ENSG00000182446	NPL4 homolog, ubiquitin recognition factor
7	CIT	1	11113	ENSG00000122966	citron rho-interacting serine/threonine kinase
8	RFX8	1	731220	ENSG00000196460	RFX family member 8, lacking RFX DNA binding domain
9	SSX3	1	10214	ENSG00000165584	SSX family member 3
10	CDC45	1	8318	ENSG00000093009	cell division cycle 45
11	PXN	1	5829	ENSG00000089159	paxillin
12	GRPEL2	1	134266	ENSG00000164284	GrpE like 2, mitochondrial
13	ITGA5	1	3678	ENSG00000161638	integrin subunit alpha 5
14	ADAMTS14	1	140766	ENSG00000138316	ADAM metallopeptidase with thrombospondin type 1 motif 14
15	PHLDA1	1	22822	ENSG00000139289	pleckstrin homology like domain family A member 1
16	RPSAP58	1	388524	#N/A	ribosomal protein SA pseudogene 58
17	SEMA3A	1	10371	ENSG00000075213	semaphorin 3A
18	NOSTRIN	1	115677	ENSG00000163072	nitric oxide synthase trafficking
19	ADM	-1	133	ENSG00000148926	adrenomedullin
20	GTF2IRD2B	-1	389524	ENSG00000174428	GTF2I repeat domain containing 2B
21	ITGBL1	1	9358	ENSG00000198542	integrin subunit beta like 1
22	B3GNT1	-1	#N/A	#N/A	#N/A
23	QDPR	1	5860	ENSG00000151552	quinoid dihydropteridine reductase
24	KIF4A	1	24137	ENSG00000090889	kinesin family member 4A
25	BLM	1	641	ENSG00000197299	Bloom syndrome RecQ like helicase
26	ZNF471	1	57573	ENSG00000196263	zinc finger protein 471
27	CADPS	1	8618	ENSG00000163618	calcium dependent secretion activator
28	SLC20A1	1	6574	ENSG00000144136	solute carrier family 20 member 1
29	SKA3	1	221150	ENSG00000165480	spindle and kinetochore associated complex subunit 3
30	CKAP2L	1	150468	ENSG00000169607	cytoskeleton associated protein 2 like
31	RPL39L	1	116832	ENSG00000163923	ribosomal protein L39 like
32	ICOSLG	1	23308	ENSG00000160223	inducible T cell costimulator ligand
33	CDK2AP1	1	8099	ENSG00000111328	cyclin dependent kinase 2 associated protein 1

34	IL20RB	1	53833	ENSG00000174564	interleukin 20 receptor subunit beta
35	EIF4E3	1	317649	ENSG00000163412	eukaryotic translation initiation factor 4E family member 3
36	GTF2IRD2	1	84163	ENSG00000196275	GTF2I repeat domain containing 2

Oncogene Signature

	Symbol	Direction	Entrez ID	Ensemble ID	Description
1	COL5A2	1	1290	ENSG00000204262	collagen type V alpha 2 chain
2	ECM1	1	1893	ENSG00000143369	extracellular matrix protein 1
3	NREP	1	9315	ENSG00000134986	neuronal regeneration related protein
4	MPP3	1	4356	ENSG00000161647	membrane palmitoylated protein 3
5	PXDN	1	7837	ENSG00000130508	peroxidasin
6	ANXA8	1	653145	ENSG00000265190	annexin A8
7	CLOCK	1	9575	ENSG00000134852	clock circadian regulator
8	MYL9	1	10398	ENSG00000101335	myosin light chain 9
9	LOC91316	1	#N/A	#N/A	#N/A
10	IGFBP5	1	3488	ENSG00000115461	insulin like growth factor binding protein 5
11	BST2	1	684	ENSG00000130303	bone marrow stromal cell antigen 2
12	PMP22	1	5376	ENSG00000109099	peripheral myelin protein 22
13	KIAA1641	1	#N/A	#N/A	#N/A
14	C2	1	717	ENSG00000166278	complement C2
15	CTSK	1	1513	ENSG00000143387	cathepsin K
16	CSTB	1	1476	ENSG00000160213	cystatin B
17	FBN1	1	2200	ENSG00000166147	fibrillin 1
18	GBP1	1	2633	ENSG00000117228	guanylate binding protein 1
19	ARHGEF2	1	9181	ENSG00000116584	Rho/Rac guanine nucleotide exchange factor 2
20	NDUFB7	1	4713	ENSG00000099795	NADH:ubiquinone oxidoreductase subunit B7
21	RCN1	1	5954	ENSG00000049449	reticulocalbin 1
22	MSN	1	4478	ENSG00000147065	moesin

23	ALDOC	1	230	ENSG00000109107	aldolase, fructose-bisphosphate C
24	EPS8	1	2059	ENSG00000151491	epidermal growth factor receptor pathway substrate 8
25	CLIC1	1	1192	ENSG00000213719	chloride intracellular channel 1
26	KLF5	1	688	ENSG00000102554	Kruppel like factor 5
27	HIST2H2AA3	1	#N/A	#N/A	#N/A
28	LAMA4	1	3910	ENSG00000112769	laminin subunit alpha 4
29	C5	-1	727	ENSG00000106804	complement C5
30	BCAT1	-1	586	ENSG00000060982	branched chain amino acid transaminase 1
31	SSFA2	-1	6744	ENSG00000138434	sperm specific antigen 2
32	LOXL1	-1	4016	ENSG00000129038	lysyl oxidase like 1
33	MT1X	-1	4501	ENSG00000187193	metallothionein 1X
34	ALDH1A1	-1	216	ENSG00000165092	aldehyde dehydrogenase 1 family member A1
35	ATP5S	-1	27109	ENSG00000125375	ATP synthase, H+ transporting, mitochondrial Fo complex subunit s (factor B)
36	LMF2	-1	91289	ENSG00000100258	lipase maturation factor 2
37	EPHX2	-1	2053	ENSG00000120915	epoxide hydrolase 2
38	F11	-1	2160	ENSG00000088926	coagulation factor XI
39	CYBA	-1	1535	ENSG00000051523	cytochrome b-245 alpha chain

Strat Signature

	Symbol	Direction	Entrez ID	Ensemble ID	Description
1	SIGLEC11	1	114132	ENSG00000161640	sialic acid binding Ig like lectin 11
2	KLF6	1	1316	ENSG00000067082	Kruppel like factor 6
3	FOSB	-1	2354	ENSG00000125740	FosB proto-oncogene, AP-1 transcription factor subunit
4	ATP4A	1	495	ENSG00000105675	ATPase H+/K+ transporting alpha subunit
5	NFKBIZ	1	64332	ENSG00000144802	NFKB inhibitor zeta
6	GSG1	-1	83445	ENSG00000111305	germ cell associated 1
